# Supplementary material for: Genome of the early spider-orchid Ophrys sphegodes provides insights into sexual deception and pollinator adaptation
Source: Nat Commun. 2024 Jul 26;15:6308. doi: 10.1038/s41467-024-50622-4 (PMC11282089; doi:10.1038/s41467-024-50622-4)
Supplement: Supplementary file 1 — Supplementary Information [file 41467_2024_50622_MOESM1_ESM.pdf]

Genome of the early spider-orchid *Ophrys sphegodes* provides insights into sexual deception and pollinator adaptation

Russo et al.

Supplementary Information

Supplementary Method 1. Genome assembly

Genome assembly and long read polishing

Nanopore long reads were base-called with Guppy v3.0.4[1], and their quality assessed with NanoPlot v1.0.0[2] (Supplementary Fig. 3). All reads were filtered using Filtlong v0.2.0 (https://github.com/rrwick/Filtlong) with the options `--min_length 10000 --target_bases 125000000000` to discard all reads shorter than 10 000 bp and remove low quality reads until 125 Gb remained. To generate the genome assembly, filtered reads were used as input to run Minimap2 v2.17 and Miniasm v0.3[3] (with options `-R -c 2`). Initial contigs were polished with long-read data, first by mapping the long reads against the assembly with Minimap2, then using the mapped reads and the draft assembly as input for Racon v1.4.3[4] (parameters `--match 8 --mismatch -6 --gap -8 --window-length 500`). This polishing process was iterated four times. Then, a consensus sequence was created with the medaka_consensus module (medaka v0.11.4; https://github.com/nanoporetech/medaka) (parameters `--model r941_prom_high_g303`) .

Illumina sequencing and short read polishing

Genomic DNA extracted for Nanopore sequencing (individual SPH_8 from the Gargano region, South Italy) was sheared using a Covaris sonicator, then used to prepare two size-selected DNA libraries with the TruSeq DNA Nano kit (Illumina). Libraries were sequenced on an Illumina NovaSeq 6000 platform (2× 150 nt and 2× 250 nt; Supplementary Table 3). Four additional libraries were prepared from the individual SPH_511B (also from the Gargano region), with the TruSeq DNA Nano Kit (Illumina), and then sequenced on Illumina HiSeq 2500/4000 (2× 150 nt; Supplementary Table 3). The NovaSeq sequence reads were trimmed using Cutadapt v2.8[5], with the following parameters for adaptor removal: `--minimum-length 1 --trim-n -a GATCGGAAGAGCACACGTCTGAACTCCAGTCAC -A AGATCGGAAGAGCGTCGTGTAGGGAAAGAGTGT`. The HiSeq sequence reads were trimmed to remove poor-quality reads, using: `--quality-base 20 -minimum-length 1` (only one dataset needed trimming at both ends, specified with `--quality-base 28,15`). Illumina short reads were mapped against the assembly using BWA-MEM v2.1[6], and the resulting bam files and the assembly (after the medaka step) were used as input for Pilon v1.23[7] to further polish the assembly with short reads. This process was repeated three times in total: the first and second round were performed with the NovaSeq dataset (individual SPH_8) with parameters `--fix all`. A third round was performed including the

HiSeq dataset (individual SPH_511B) and parameter `--fix indels`, to fix small indels with short reads from another individual. A schematic representation of the assembly strategy is provided in Supplementary Fig. 4.

Heterozygosity in the genome and gap closing

Prior to genome assembly, the overall heterozygosity rate of the *O. sphegodes* genome was estimated based on the *k*-mer count distribution with GenomeScope (http://qb.cshl.edu/genomescope/) and Illumina reads. The model calculates the heterozygosity rate based on duplicated *k*-mers from maternal and paternal origin and the relative heights of the homozygous and heterozygous peaks, and revealed a heterozygosity rate of 1.28% (meaning that the heterozygous peak matches the height of the homozygous peak at around 1.28 % for k = 21) (Supplementary Fig. 2). The assembly of heterozygous genomes is a challenging task because it is difficult to accurately differentiate between heterozygous haplotypes[8–11]. When two heterozygous sequences of the same region exceed a certain threshold of nucleotide diversity, the genome assembler will consider them as separate contigs, i.e., sequences belonging to different regions of the genome. Instead of being collapsed as single-haplotype contig, or discarded, these sequences are included as extra contigs in the genome assembly, thus inflating assembly size[12,13]. Furthermore, these extra contigs make the assembly more fragmented, as gaps between sequences cannot be solved when two possible alternative solutions are present[14]. In the case of the *Ophrys* genome, after assembling and polishing the contigs, the assembly size accounted for 6.4 Gb, more than 1 Gb larger than the estimated genome size. To remove under-collapsed heterozygous contigs, therefore, the Redundans v0.11 pipeline[15] was used (Supplementary Fig. 4). The pipeline considers the similarity between two sequences and read coverage depth to detect and remove redundant contigs (used with parameters `-b -m 400 --identity 0.51 --overlap 0.80`). Then, short and long reads are used by Redundans for gap closing (2 iterations). Final assembly size (Osph-v1.0.fa) at this stage was 5.2 Mb, with a total of 8 074 contigs (Supplementary Table 4).

Hi-C library preparation and genome assembly scaffolding

To scaffold the genome assembly to chromosomes, an *in situ* Hi-C library was prepared as described previously[16] with modifications[17]. Individual SPH_2 from Capoiale, Italy, was

chosen for the experiment. Briefly, 0.5 g of leaf tissue was treated with nuclei isolation buffer and 36% formaldehyde in a vacuum pump, to extract cell nuclei and cross-link chromatin. Plant material was then crushed with mortar and pestle in liquid nitrogen, centrifuged to extract the supernatant (nuclei), digested with the enzyme *Hin*dIII-HF (New England Biolabs, Ipswich, MA, USA, R3104L), and re-ligated for cross-link reversal. DNA was then extracted by phenol:chloroform:isoamyl alcohol (25:24:1) and cleaned up. Four Hi-C libraries were prepared with the KAPA HyperPrep Kit (Roche, Switzerland, KK0502), and sequenced on an Illumina NovaSeq 6000 SP FlowCell (2× 150 nt) to produce a total of 1 billion reads (Supplementary Table 3). To assemble the chromosomes, the *de novo* assembly and Hi-C reads were used as input for a combined approach including different pipelines. First, raw Hi-C data was mapped against the genome assembly with the ArimaGenomics mapping script, specifically developed for Hi-C short reads (https://github.com/ArimaGenomics/mapping_pipeline/blob/master/01_mapping_arima.sh). Then, the bam file was used as input for building scaffolds with Salsa v2.3[14,18] (parameters: `--enzyme AAGCTT --clean yes --cutoff 1000`). After Salsa, 2 520 scaffolds remained (Osph-v1.2.fa). Finally, scaffolds were linked together to chromosomes using Juicebox Assembly Tools (JBAT)[19] and the 3D-DNA pipeline v-180114[20]. First, raw Hi-C short reads were mapped against the assembly version Osph-v1.2.fa with Juicer v-1.5.6 (parameters: `-s HindIII -C 40000000 -D early`). Then, final chromosomes were assembled by the 3D-DNA pipeline (parameters: `--input 20000 --editor-coarse-resolution 500000 --editor-coarse-region 2000000 --editor-fine-resolution 25000 --editor-coarse-stringency 40 --editor-repeat-coverage 5`), and manual curation of the chromosomes was performed with the visualisation tool embedded in JBAT. As a last step, HiC-Hiker[21] v1.0.0 was used to further reduce scaffold misorientation at small scale, with option `-K 90000`, to produce the final *O. sphegodes* genome assembly (Osph-v2.3.fa, Supplementary Table 4).

Quality assessment and validation

The completeness and quality of the final *O. sphegodes* assembly was assessed with three methods. First, 267 billion Illumina paired-end reads (Supplementary Table 3) were mapped to the genome using BWA-MEM2 v2.1[6] with default parameters. In total, 97.8% of the reads could be mapped to the genome overall and 85.9% were properly paired (Supplementary Table

6). Then, one PacBio library was prepared from DNA extracted from the same individual (SPH_8), as previously described[22]. This library was used to sequence 2 SMRTcells on a PacBio Sequel system, chemistry 3.0. The resulting total reads (1 533 673, 2× coverage) were mapped to the genome with minimap 2.17 and default parameters. This resulted in a total mapping percentage of 98.06% (95.07% primary mappings). Furthermore, BUSCO v5.1.3[23,24] was run in genome mode using the embryophyta_odb10 database (release 2020-09-10) based on 1 614 universal single-copy orthologues. In total, 1 369 (84.9%) ultra-conserved genes were found in full length (complete) and 100 (6.2%) were found to be partial (fragmented) in the final assembly. The overall completeness of the final *O. sphegodes* assembly estimated based on BUSCO is similar to the *V. planifolia* haplotype A genome, and only slightly less than other orchid genomes (except for *G. elata*) (Supplementary Fig. 6a). A comparison of the genome's BUSCO results with the ones of the *O. sphegodes* transcriptome[25] shows consistent 'complete' and 'missing' values (BUSCO on *O. sphegodes* transcriptome run with embryophyta_odb10 results: Complete, 88.3%; Fragmented, 4.4%; Missing, 7.3%). Taken together, these analyses suggest that the overall quality of the assembly is high.

Supplementary Method 2. Chromosome-wide DNA Nanopore methylation map

Nanopore sequencing can detect methylated cytosine (mC) in plants, as they provide a characteristic raw electric signal compared to unmethylated cytosine[52]. To measure 5-mC, sequencing data in FAST5 were basecalled with Guppy v5.0.11[1] with option `-c dna_r9.4.1_450bps_modbases_5mc_hac_prom.cfg -device cuda:0`. Guppy uses a deep learning recurrent neural network (RNN) model on the nanopore current signal to distinguish 5-mC from unmethylated cytosine (https://community.nanoporetech.com/technical_documents/). The methylation caller generates a log-likelihood value for the probability of methylated to unmethylated cytosines. The newly called fastq files were merged into a single one and indexed, to relate the Nanopore electric signal data to read IDs (`nanopolish index -s sequencing_summary.txt`). The fastq reads were mapped to the reference genome with Minimap2, with option -`ax map-ont --split-prefix string`. Then, Nanopolish v0.13.3 (https://github.com/jts/nanopolish) was used to detect methylated bases with option `call-methylation -r file.fastq -b mapping.bam -g genome.fa`. The result was a .tsv table with

position of the CG dinucleotide (start/end), read ID, sequence, log-likelihood ratio of methylated/unmethylated base, where a positive value indicated support for methylation. Finally, methylation frequency was calculated with the calculate_methylation_frequency.py script provided in the nanopolish toolkit, using a log-likelihood ratio greater than 1.5 (methylated) as threshold for calling methylation[53]. Supplementary Fig. 7 and 8 show methylation count per chromosome.

Supplementary Method 3. Genome annotation

TE annotation

Transposable elements (TE) were manually characterised according to the method described in ref.[26]. First, putative TEs were identified with RepeatModeler v2.0.1 and RepeatMasker v4.1.0[27], and then blasted against chromosome 1 of the *O. sphegodes* genome. Resulting sequence hits were retrieved from the genome fasta file with their flanking regions (6 000 nt to 10 000 nt upstream and downstream, depending on the sequence class), and used as input for a multiple sequence alignment (MSA) with CLUSTAL W[28]. Resulting alignments were visually inspected to identify start and end motifs and to characterise the consensus sequence. All consensus sequences were renamed according to the three-letter code described in ref.[26]. This procedure was employed for all putative repeats originally pre-assigned by RepeatMasker as part of a superfamily. Unfortunately, ~30% repetitive elements were classified as unknown by RepeatMasker. For those, the putative consensus sequences obtained from the MSA were blasted against the TRansposable Elements Platform (TREP) database (v. 20Aug2019, available at https://trep-db.uzh.ch/), and PTREP (Protein TRansposable Elements Platform, release 19) and Dfam (https://dfam.org), and then revised accordingly. If the unknown sequence did not have any match in any of the databases, we manually examined the MSA for presence of structural motifs that could help to characterise and classify the TE. By following this approach, we were able to characterise a total of 436 novel TEs in *O. sphegodes,* and created the first curated TE database of the Orchidaceae family. This data set has now been included in the TREP database (available at https://trep-db.uzh.ch/). Finally, we used this species-specific library to annotate the TEs in the genome with RepeatMasker v4.1.0 (`-engine rmblast -nolow -norna -no_is`).

Annotation of protein coding genes

The soft-masked *O. sphegodes* genome was annotated using a combined approach that included BRAKER2 v2.1.6[29] with AUGUSTUS v3.4.0[30], GeneMark-EX v4.64_lic[31] with ProtHint v2.6.0, and DIAMOND v0.9.24[32], for *de novo* gene prediction; GeMoMa v1.8[33] for homology-based prediction; PASA v2.5.1[34] to model gene structures based on transcriptome evidence, annotate UTRs and deal with alternative splicing variants. First, protein hints were generated by ProtHint using the protein database liliopsida_odb10 v.2020-09-10 from OrthoDB10, to score intron intervals, start and stop codons from ultra-conserved proteins of the monocots lineage. This extrinsic evidence was given to GeneMark for self-training. AUGUSTUS was specifically trained for *O. sphegodes* using RNA-seq data from floral tissues of *O. sphegodes* and the three closely related species: *O. exaltata, O. incubacea, O. garganica* (previously published[25], available under SRA accession PRJNA574279). First, all RNA-seq fastq were mapped against the unmasked genome assembly with STAR v2.7.5c[35] (parameters: `--readQualityScoreBase 28 -outFilterScoreMinOverLread 0  -outFilterMismatchNmax 2`). Then, the bam files were used as input for the BRAKER2 pipeline to train AUGUSTUS. A second BRAKER2 run was performed with `-etpmode` and hints from the first run, to improve the prediction accuracy of AUGUSTUS and include predictions from GeneMark. The resulting gff file was used as an input for GeMoMa, for gene prediction based on homology with the genome annotations of *Phalaenopsis equestris*[36], *Cymbidium goeringii*[37], *Oryza sativa* ssp. *japonica* cultivar Nipponbare IRGSP-1.0[38] and *Asparagus officinalis* Aspof.V1[39]. PASA was run on the final prediction to model gene structures based on the transcriptome assembly of *O. sphegodes s.l.*[25]. First, the transcriptome was mapped against the genome assembly with Minimap2, and alignments were clustered and assembled. Then, the PASA assemblies were incorporated into our final gene predictions to correct exon boundaries, add UTRs, and update gene structures. At this stage, the total number of gene models was 155 025.

The predicted gene models were filtered based on their repeat content (after TE annotation). All genes with a repeat content ≥40% of their length were removed. Gene models were also removed if the exact same gene structure was predicted multiple times independently by different algorithms. Moreover, when different isoforms of the same gene were predicted as different genes, they were clustered together into the same gene structure. At this stage, the

total number of coding sequences (CDS) was 136 555. An extra filtering step was carried out based on RNA-seq support, i.e., genes with little RNA-seq evidence or no functional annotation were removed from downstream analysis (keeping genes with RNA-seq TPM ≥ 5). All gene models were integrated in AHRD v3.3.3 (Automated assignment of Human Readable Descriptions), that classifies predicted sequences based on bit score of the blast results against SwissProt, TAIR10, trEMBL, overlap score, and token score of assigned Human Readable Description, where tokens are sequences of characters with a human readable meaning. We used AHRD with default parameters and considered valid only those genes that met the following three criteria: bit score > 50 with e-value < e-10; blast overlap > 60%; token score > 0.5. Genes with premature stop codons were considered to be pseudogenes and were removed from the final list. The final number of CDS after this filtering step was 42 542.

Candidate genes for hydrocarbon biosynthesis[40–43] and other traits of interest were also included in our annotation. This was done semi-manually for each candidate gene and then manually curated. Coding sequences of a priori candidate genes were used in BLASTN searches against the genome assembly (both reference and alternative heterozygous haplotigs, i.e., contigs discarded from the final assembly via Redundans) using an e-value of 1E-15. Hits were filtered by percentage of sequence identity (typically between 90 and 95%) using varying threshold values reflecting phylogenetic distance between *Ophrys* and the organism of sequence origin. Valid hits were visualised in R to discern consecutive areas of homology along an *Ophrys* contig, putatively corresponding to different exons. When a homologue was found, the corresponding contig sequence was extracted from the lowest to the highest BLASTN hit coordinates plus flanking regions (typically of 5kb), and the contig aligned to the query CDS using est2genome from the EMBOSS 6.6.0.0 package (emboss.sourceforge.net)[44]. Resulting alignments were manually inspected in BioEdit 7.2.6[45], especially putative exon/intron boundaries, to obtain putative gene models maximally consistent with those previously described. All manually curated genes were included in the final gene prediction. In case a manually annotated gene overlapped with an automatically predicted one, we chose to keep the manual version. Supplementary Data 1 contains the complete list of manually annotated candidate genes; *SAD* genes and transcription factors in this set are shown in Supplementary Tables 7 and 8, respectively.

Functional annotation of protein coding genes

The function, gene ontology (GO), and protein domain of filtered predicted coding genes were annotated with TRAPID 2.0[46]. Protein coding sequences were first compared to the PLAZA 4.5 monocots database[47] for similarity search, using Orchidaceae as phylogenetic clade and DIAMOND to blast query sequences against the database with e-value cut-off of 1E-5. After initial processing, the InterPro database v.87.0 was used for Protein Domain search. In total, 38 978 transcripts were assigned to at least one GO term, 38 845 had at least one Protein Domain. The analysis also grouped predicted transcripts in 7 154 gene families. The top three gene families were HOM04x5M000001, with 774 transcripts, involved in protein binding; HOM04x5M000002, with 473 transcripts, with putative protein kinase activity; HOM04x5M000003, with 251 transcripts, with putative oxidoreductase activity. KEGG annotation was performed with KAAS (KEGG Automatic Annotation Server; https://www.genome.jp/tools/kaas/), and enzyme commission (EC) numbers were assigned to the predicted genes. Overall, 7 978 genes were assigned to KEGG orthologues (KO). Genes annotated in this way were again screened for genes of *a priori* interest, specifically for additional genes putatively involved in hydrocarbon or very-long-chain fatty acid (VLCFA) biosynthesis, anthocyanin biosynthesis and carotenoid biosynthesis (Supplementary Data 2).

Annotation of transcription factors and protein kinase genes

Transcription Factors (TF) and Protein Kinase (PK) genes were identified using the iTAK Classifier (http://itak.feilab.net/cgi-bin/itak/index.cgi)[48]. A total of 2 078 coding genes were identified as transcription factors or transcriptional regulators, and 917 genes were identified as protein kinase (Supplementary Data 3 and 4). MADS-box genes were further assigned to gene groups using the pipeline described in ref.[49].

Annotation of tRNAs and rRNAs

The structure and number of transfer RNAs were predicted using tRNAscan-SE v2.0.9[50] and Infernal 1.1.4[51]. A total of 2 239 candidate tRNA genes were found, of which 1 608 were confirmed by Infernal. Of these, 1 238 tRNA decode codons of the standard 20 amino acids, 9 are possible suppressor tRNAs, and 15 have undetermined isotypes; 346 were likely

pseudogenes. Barrmap v0.9 (https://github.com/tseemann/barrnap) was used to annotate ribosomal RNA with default parameters and `--kingdom euk`. A total of 1 255 rRNA genes were found.

Supplementary Method 4. Population genetics and gene expression analyses

GBS and RNA-seq variant calling and data filtering strategy

Different data types were used for population genomic analysis, including (i) a genotyping-by-sequencing (GBS) dataset[54], and (ii) an RNA-seq data set[25]. The samples were previously collected across five collection spots in the Gargano area, south Italy, and included the following closely related species: *O. sphegodes* Miller, *O. exaltata* subsp. *archipelagi* (Gölz & H.R. Reinhard) Del Prete, *O. garganica* Nelson ex O. & E. Danesch, *O. incubacea* Bianca[55]. They are pollinated by *Andrena nigroaenea* (Kirby 1802), *Colletes cunicularius* (Linnaeus 1761), *Andrena pilipes* Fabricius 1781 (syn. A. carbonaria), *Andrena morio* Brullé 1832, respectively[41,56,57]. The different data sets had varying numbers of biological individuals vs sequencing depth (and thus genotyped variants) per individual (RNA-seq variant calling and data filtering strategy; see Supplementary Table 14). Data filtering of raw variant data in VCF format was carried out using SPA v0.1 (https://peb.uni-hohenheim.de/SPA), and putatively mis-assigned individuals were removed; the accession INC_527I was excluded from the GBS dataset, because its species assignment was unclear. Likewise, any samples with unclear population assignment and bud samples (pooled from different individuals)[25] were removed from the RNA-seq dataset. The choice of variant filtering was designed to balance needs for genotyping quality and number of diploid, biallelic markers available for analysis, making sure each individual genotype call had a >99% chance of detecting both alleles given the number of reads (n, given in the sample depth of coverage field, DP, in a VCF file) and the average read quality (q), assuming (an unrealistically low) mean read quality of *phred* score Q=10 (yielding the probability q that a base read is correct of 0.9). The worst-case scenario for assessing if both alleles are detected within one individual occurs in homozygous state, where the probability that both alleles are correct is given by $P=1-p^{nq}-(1-p)^{nq}$, with $p=0.5$, the frequency of each allele at a heterozygous site. Additionally, both more and less stringent settings were assessed for consistency of results in exploratory analyses. For distance-based analyses,

polymorphic, biallelic variant calls passing a minimal sample DP threshold of 3 when heterozygous, or of 10 when homozygous, were used for analysis. Where seemingly homozygous variants exceeded a DP threshold of 3 (but <10), only the first allele at this variant was considered for distance analysis. For BayeScan analysis of GBS data, in addition, at least 6 allele calls had to be observed in *each* species (two for pairwise, four for global F_{ST} analysis). For BayeScan analysis of RNA-seq with high read depth, only biallelic sites present in *all* individuals at per-sample DP ≥5 were analysed. SNP state transition matrices are given in Supplementary Table 15.

Distance-based analysis

Between-individual genetic distances were calculated from data sets filtered as outlined above. Here, following ref.[54], we used the unphased diploid SNP distance described in ref.[58], allowing the inclusion of partial genotypes (in which only the first allele is evaluated, and partial combinations are evaluated as 0.5; Supplementary Table 16). Distance matrices were analysed via principal coordinate analysis (PCoA)[59]; this analysis was implemented in SPA v0.1, which includes source code from the biOP library (https://sourceforge.net/projects/biop/; see ref.[54]) for distance calculation and from FAMD v1.33[60] for PCoA. Distance analyses were carried out on biallelic variants passing the filtering steps as outlined above.

F_{ST} and population distance analysis

In order to calculate F_{ST} values for population differentiation and perform F_{ST} outlier scans, datasets were filtered and converted from VCF into BayeScan format using SPA v0.1. BayeScan v2.1 was run as described in ref.[54], including their calculation of command-line PO settings from the number of available loci. BayeScan analysis was carried out both as a 'global F_{ST}' analysis, treating each of the four species as a subpopulation, and as pairwise analyses between each possible combination of pairs of species. These analyses were carried out independently for GBS and RNA-seq datasets. Additionally, to investigate genomic similarity among *O. sphegodes* and the other closely related species, for each chromosomal block, a genetic inter-population distance was calculated and averaged across multiple loci within a 1 Mb window to identify the species with the highest and lowest genetic distance. This was

implemented using the single-locus chord distance for each variant[61], averaged into a multi-locus distance as described by ref.[62].

Differential gene expression analysis

RNA-sequencing datasets from floral tissues of *O. sphegodes* and *O. exaltata*[25] were used to perform differential gene expression analysis. First, the datasets were cleaned of low-quality reads and adaptors using Trimmomatic v0.39[63] (parameters `ILLUMINACLIP:TruSeq3-PE-2.fa:2:30:10`). Trimmed reads were mapped to the genome reference with STAR v2.7.5c[35] (parameters `--readQualityScoreBase 28 --outFilterScoreMinOverLread 0 -outFilterMatchNminOverLread 0 -outFilterMismatchNmax 2 -quantMode GeneCounts`). This resulted in an average of 81.43% uniquely mapped reads. Gene counting was performed by HTSeq v2.0.1[64] (`--stranded=no -m intersection-nonempty`). Thereafter, differential gene expression analysis was carried out in R with edgeR v3.32.1[65], to compare expression between *O. exaltata* and *O. sphegodes* mature labella considering all genes with $\log_2$ (variance) < 35. Only genes that were present in at least three samples with a minimum of 10 supporting reads were considered for the analysis. Given that the original dataset[25] contained several factors potentially affecting gene expression (species, stage, environment), we specified the experimental design matrix as `model.matrix(~ 0 + species + stage + env, data = designFactors)`. Dispersions were estimated using edgeR's functions implementing generalised linear models (GLM). Model fitting used the function `glmFit` and differential expression was assessed using the function `glmLRT` with a contrast set up via `makeContrasts (levels = experimentDesign, SvE = speciesSPH - speciesEXA)`. False discovery rate (FDR) was adjusted using the '`BH`' method with function `p.adjust`. We considered genes to be differentially expressed at FDR < 0.05. Results (TMM expression and FDR values) are shown in Fig. 3 and Supplementary Fig. 12, 14 and 19.

Supplementary Method 5. Further analyses on chromosome 2

Analysis of synonymous and non-synonymous substitutions

Variant calls (see Supplementary Method 4) derived from the RNA-seq dataset[25] were used to reconstruct alleles for all CDS of protein-coding genes annotated on chromosome 2. Non-synonymous and synonymous nucleotide diversity, denoted π_a and π_s, respectively,

were calculated across all species and for each species separately using SPA 0.1. All polymorphic CDS in the chromosome intervals of interest were then screened to identify genes with an excess in non-synonymous over synonymous change, i.e. π_a - π_s > 0, but also requiring that $\pi_{a,gene} > \overline{\pi_a}$.

Gene Onthology enrichment analysis

Gene Onthology (GO) enrichment analysis was carried on for the region on chromosome 2 with elevated F_{ST}, here defined as F_{ST} > 0.25 (coordinates 333 331 279 – 352 276 360 from the GBS dataset; 327 308 632 – 357 992 435 from the RNA-seq dataset). All genes annotated in those intervals in the genome were chosen as gene sets to test significant enrichment of GO terms. The analysis was performed on R with the package topGO v3.18 (https://bioconductor.org/packages/topGO), and statistical significance was assessed via Fisher test (function `runTest(Godata, algorithm = "classic", statistic = "fisher"`). Results are shown in Supplementary Data 6.

Supplementary Figures

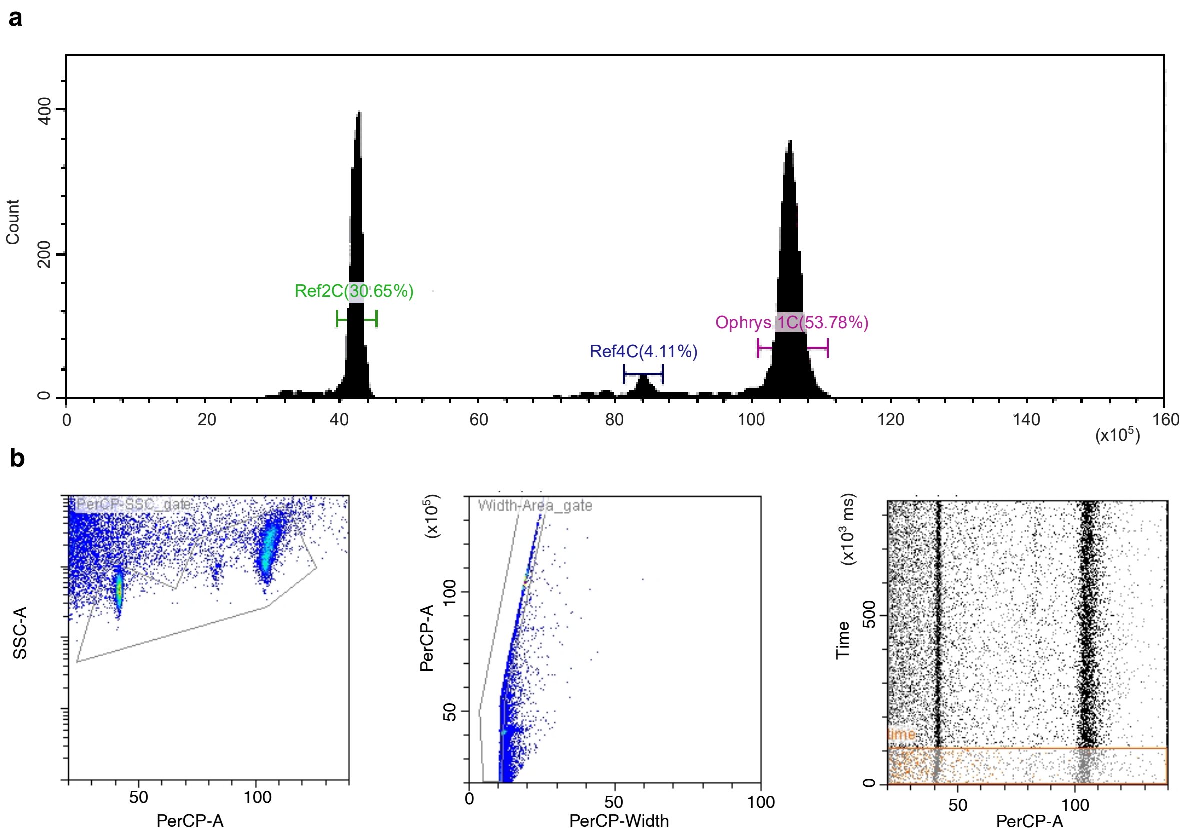

Supplementary Figure 1. Flow cytometry measurements of genome size

a. Propidium iodide fluorescence (PerCP-A) intensity of *O. sphegodes* nuclei from pollinia relative to the reference (*S. lycopersicum* leaf nuclei). The y axis shows the number of events (measured nuclei); the x axis shows relative fluorescence or light scatter intensity. Source data are provided as a Source Data file. **b.** Gating strategy used for analysis of nuclei (SSC, side scatter).

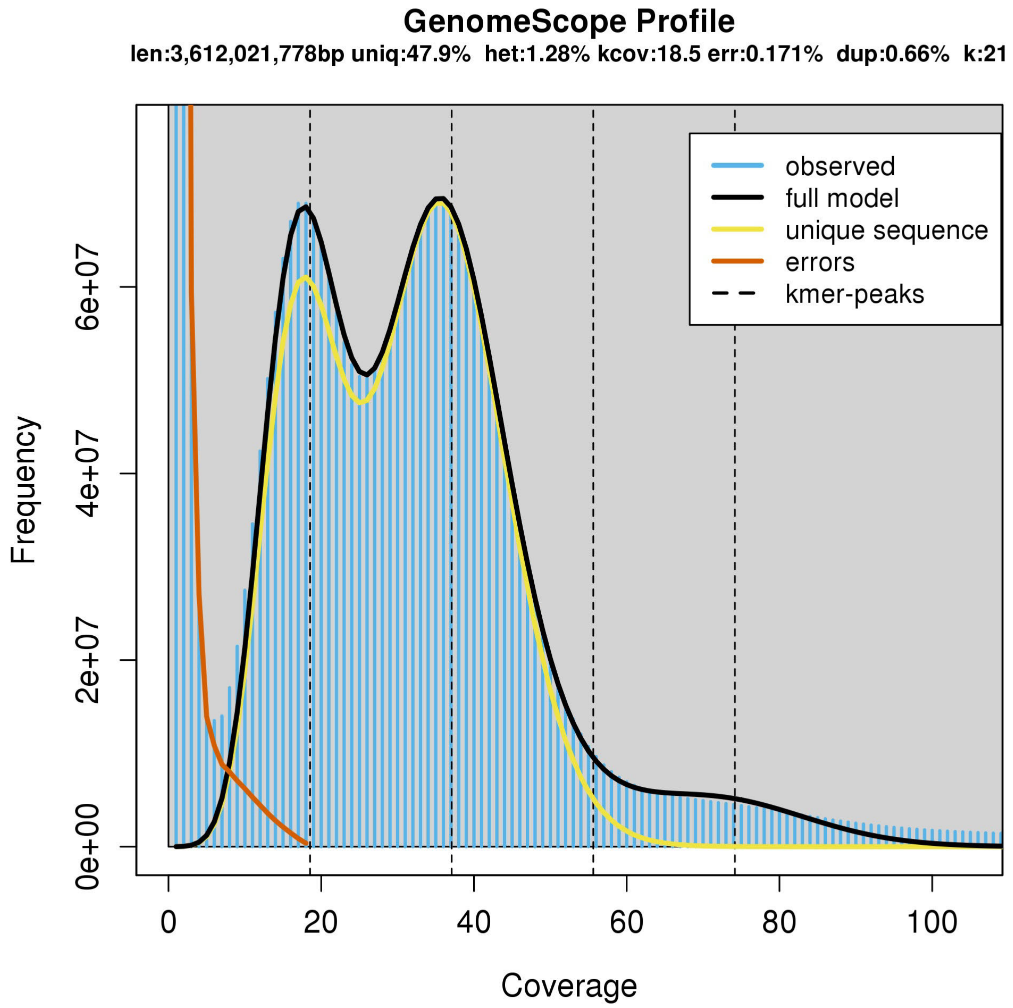

Supplementary Figure 2. Heterozygosity estimation with *k*-mer analysis

k-mer profile of *O. sphegodes* based on Illumina reads. The heterozygous portion of the genome is represented by the first peak. The homozygous portion is represented by the second peak. GenomeScope estimates the genome heterozygosity, taking into account observed *k*-mer distribution, sequencing errors, unique sequences, and fits the best *k*-mer model.

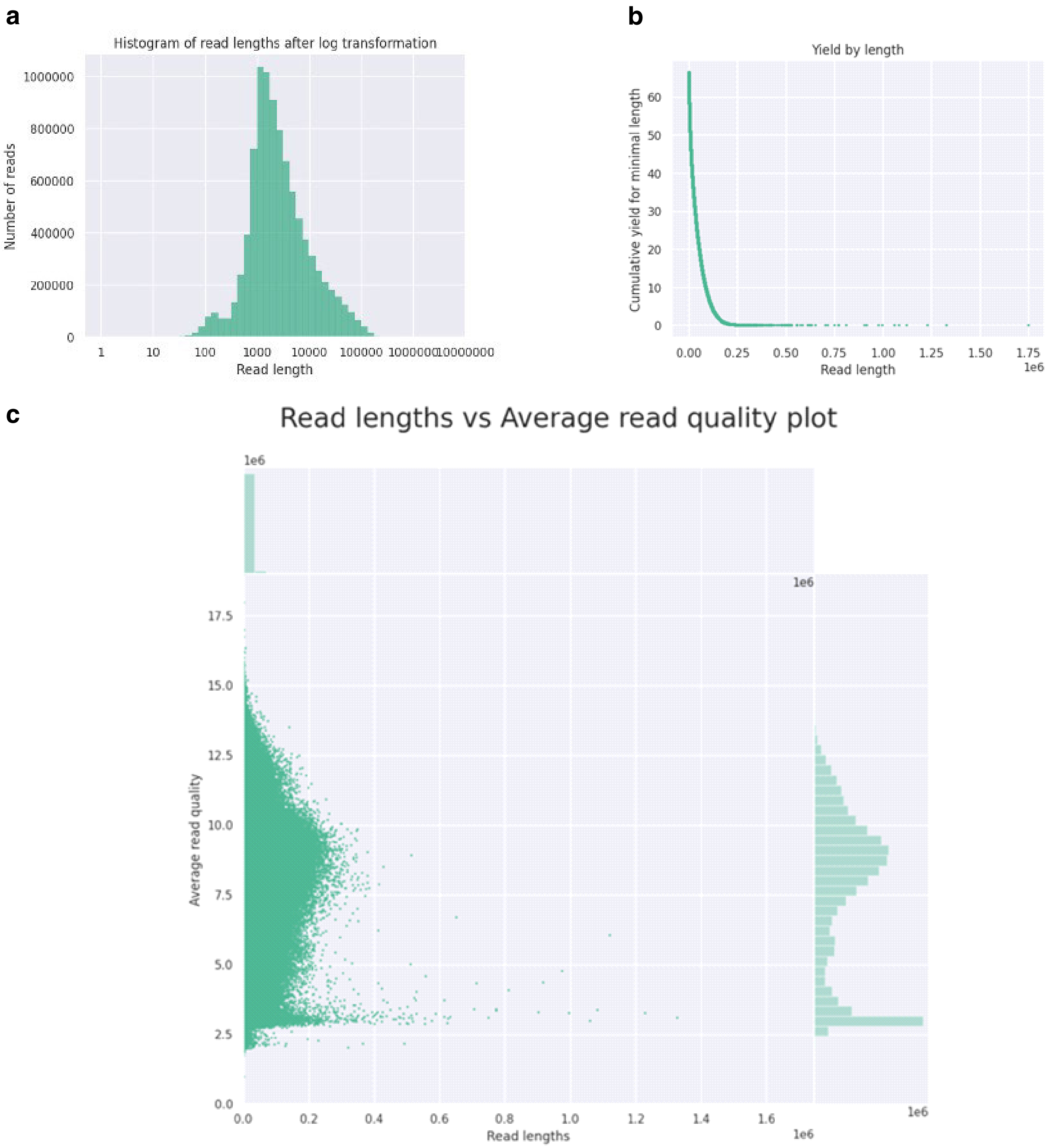

Supplementary Figure 3. Summary of sequencing statistics for the Nanopore data

Sequencing summary for PromethION dataset 4. **a**. Histogram of log-transformed read length distribution. **b**. Cumulative yield in Gb per read length. **c.** Bivariate plot of read length compared to the average read quality.

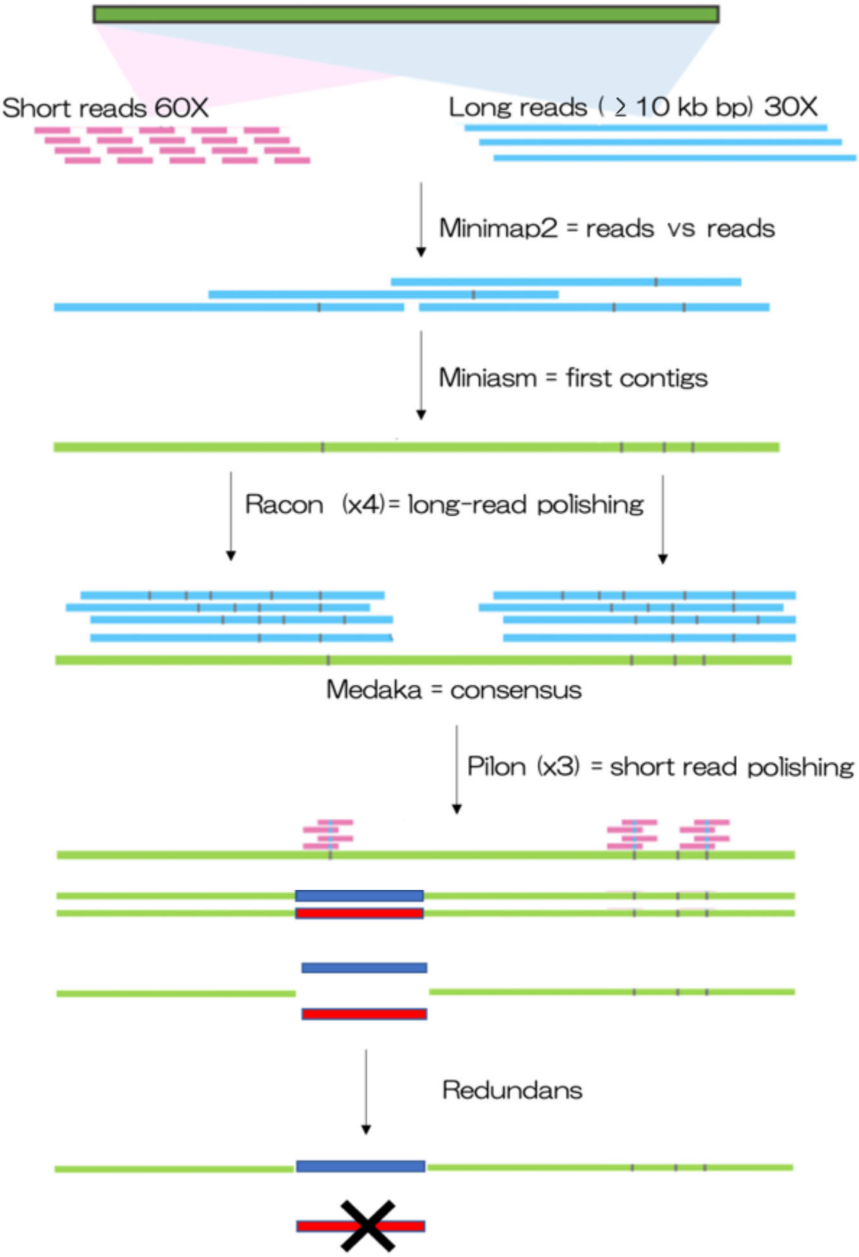

Supplementary Figure 4. Genome assembly strategy

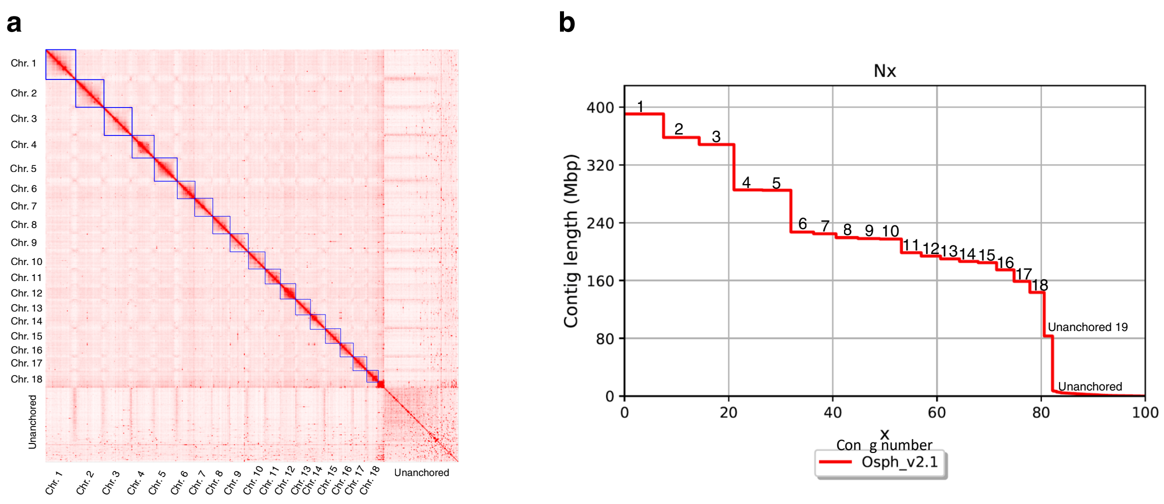

Supplementary Figure 5. Assembled chromosomes

a. Contact maps of chromosomes from Hi-C. **b.** Chromosome length in Mbp as percentage of genome length. 82.2% of the genome are contained in the first 19 scaffolds.

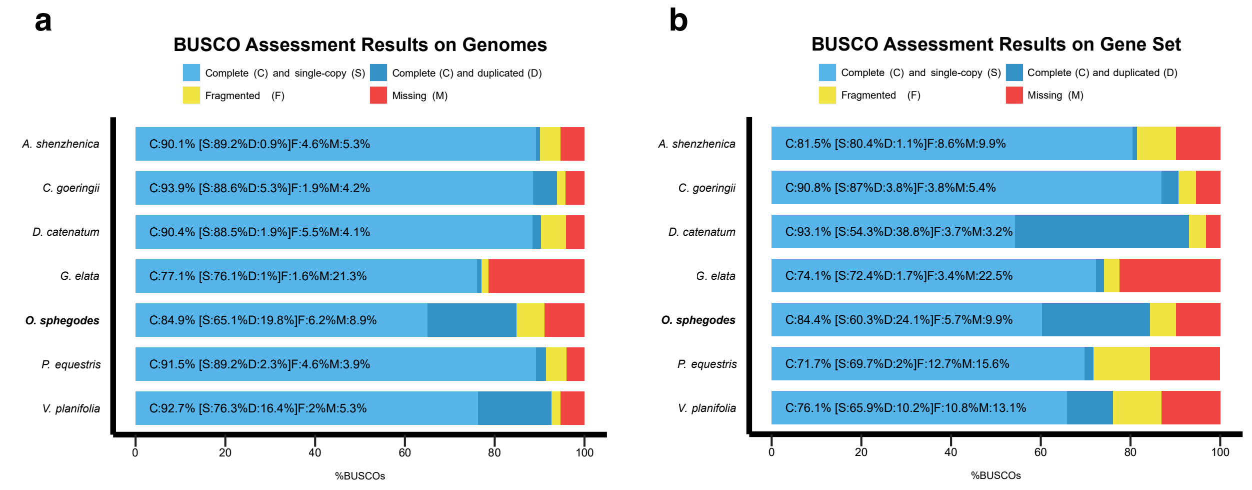

Supplementary Figure 6. Comparison of BUSCO single-copy orthologous genes across orchids

a. BUSCO results across orchid genomes. **b.** BUSCO results across orchid proteomes. BUSCO assessment was performed with the embryophyta OrthoDB_10 database.

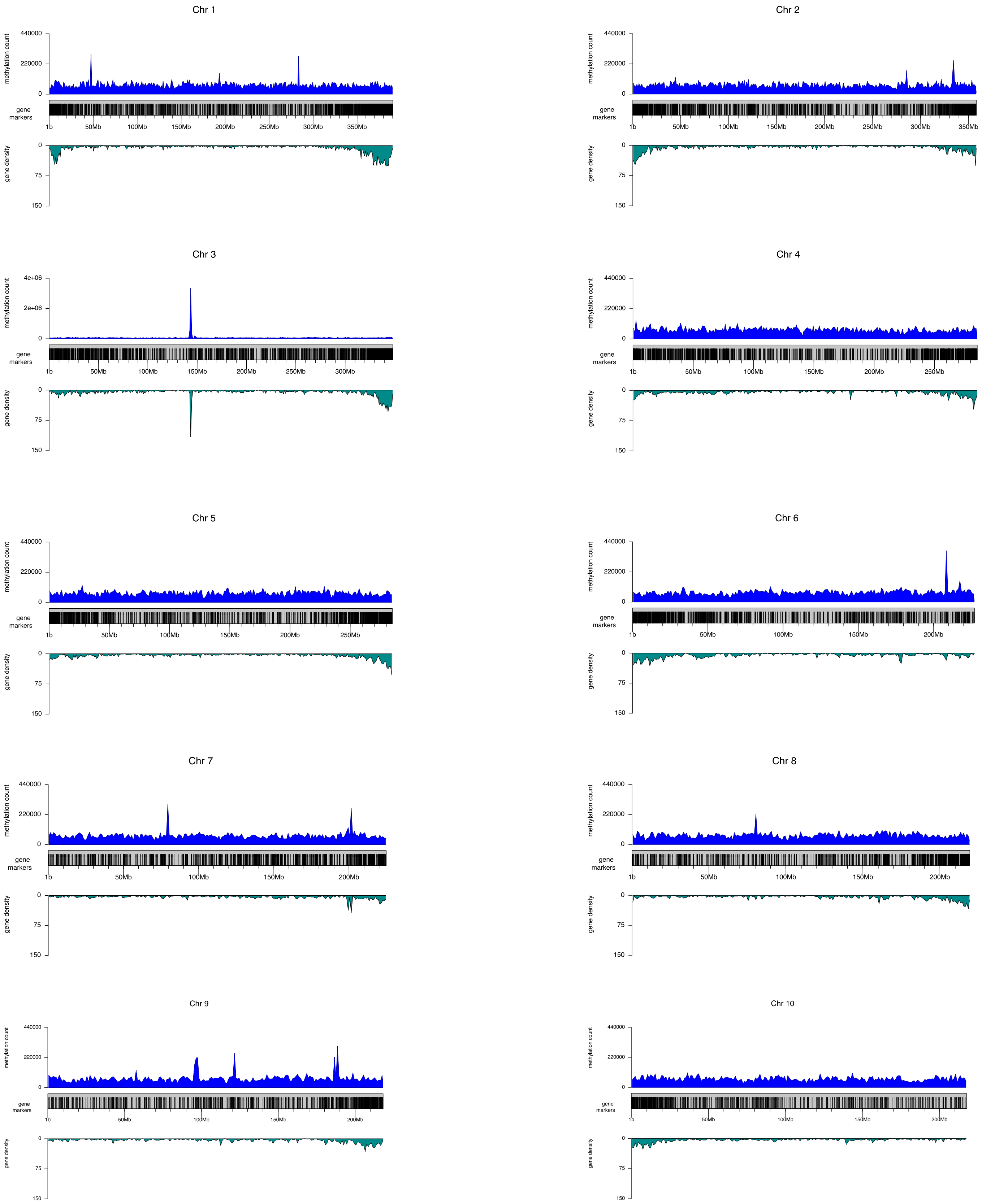

Supplementary Figure 7. Methylation patterns across chromosomes 1-10

Cytosine methylation was called from Nanopore data. Methylation count and gene density were calculated per 1Mb window and plotted along chromosomes.

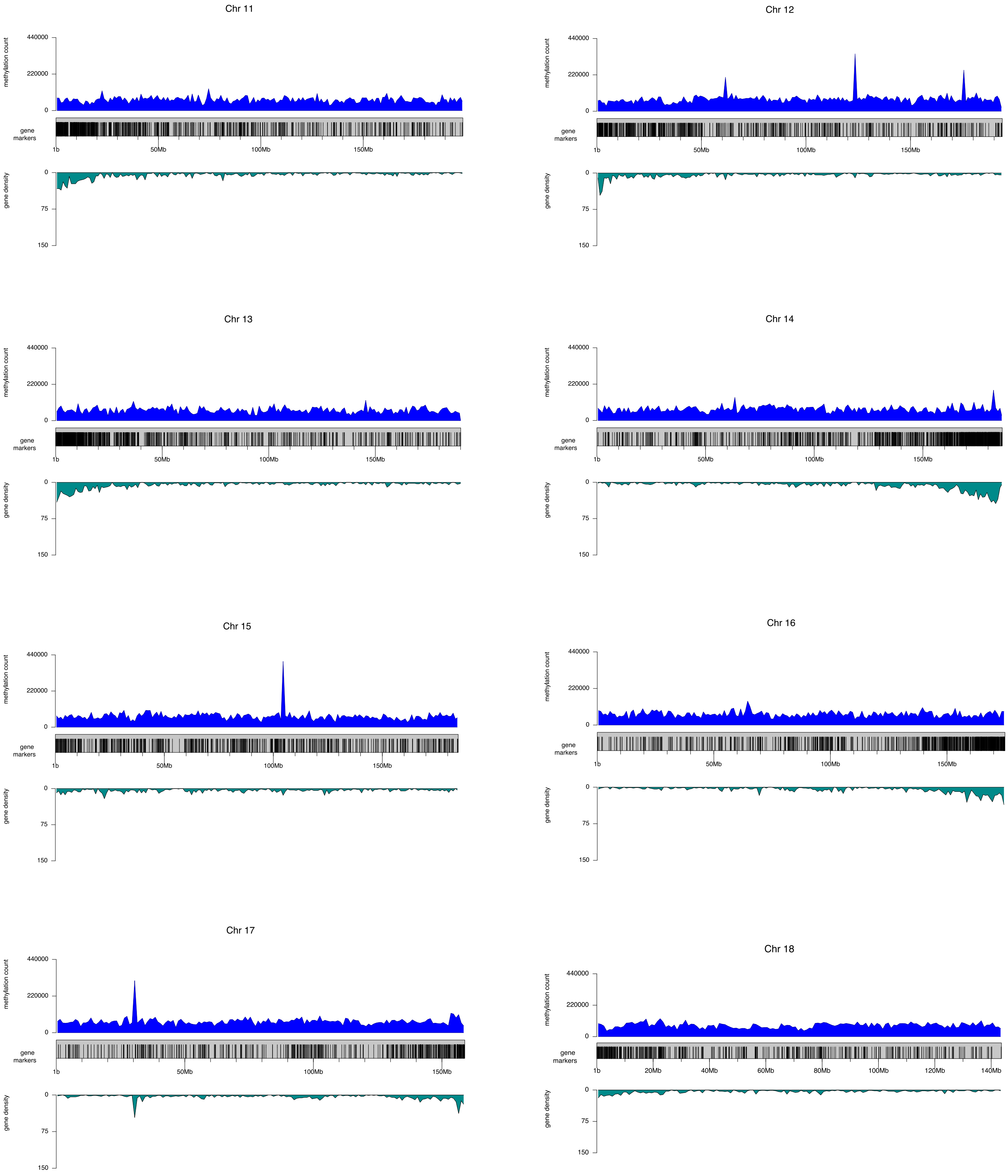

Supplementary Figure 8. Methylation patterns across chromosomes 11-18

Cytosine methylation was called from Nanopore data. Methylation count and gene density were calculated per 1Mb window and plotted along chromosomes.

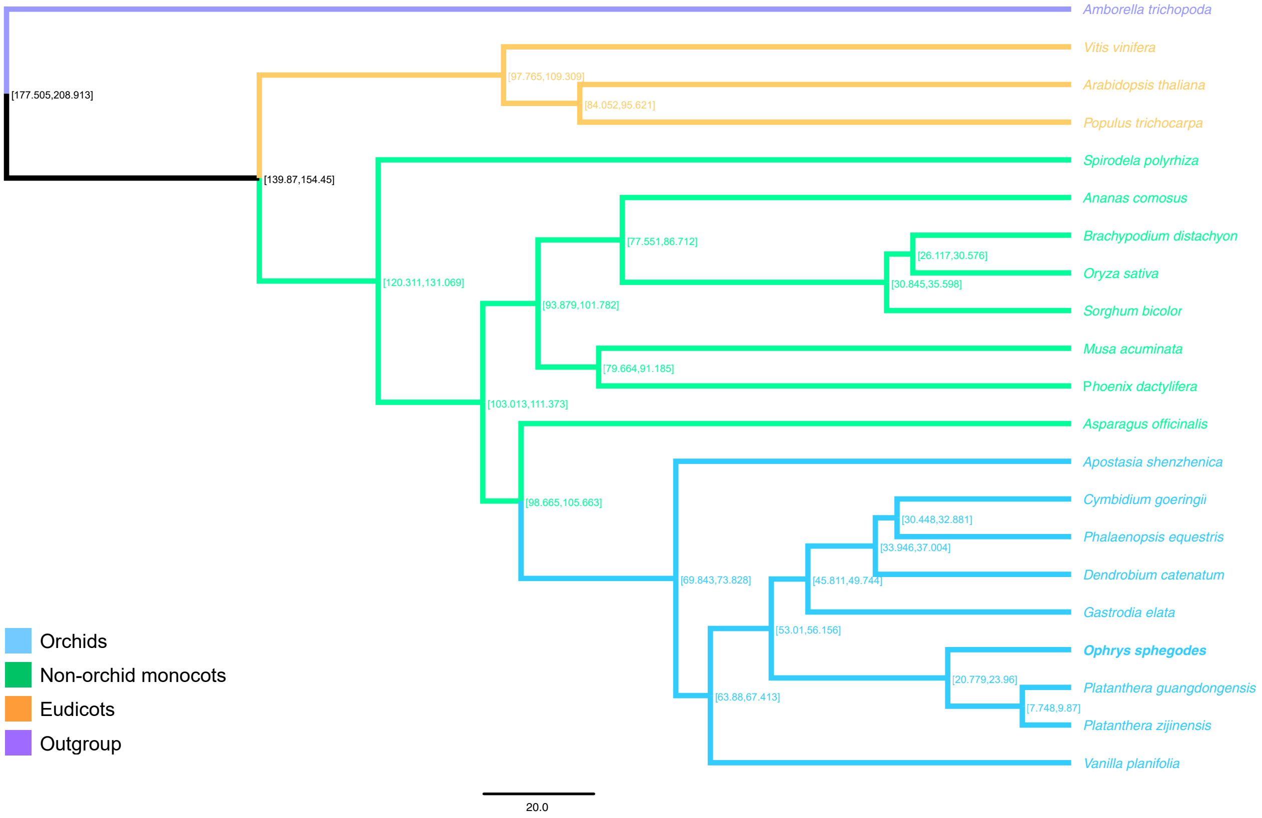

Supplementary Figure 9. Phylogenomic tree of 21 plant species showing 95% confidence intervals

Related to Fig. 2a in the main text.

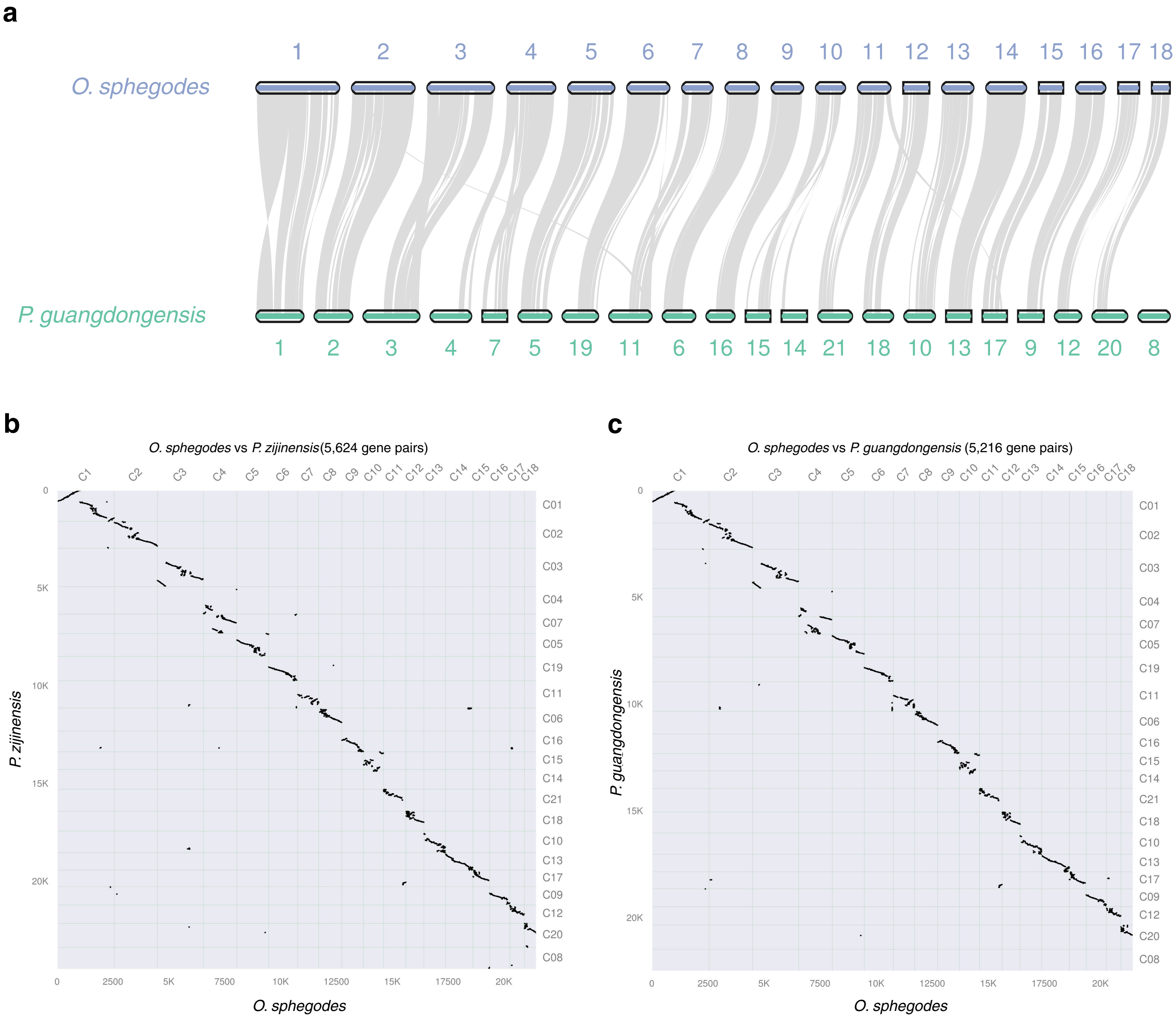

Supplementary Figure 10. Synteny analysis

a. Synteny analysis between *O. sphegodes* and *P. guangdongensis*. **b** and **c** show collinearity analysis between *O. sphegodes* and *P. zijinensis* and *P. guangdongensis*, respectively.

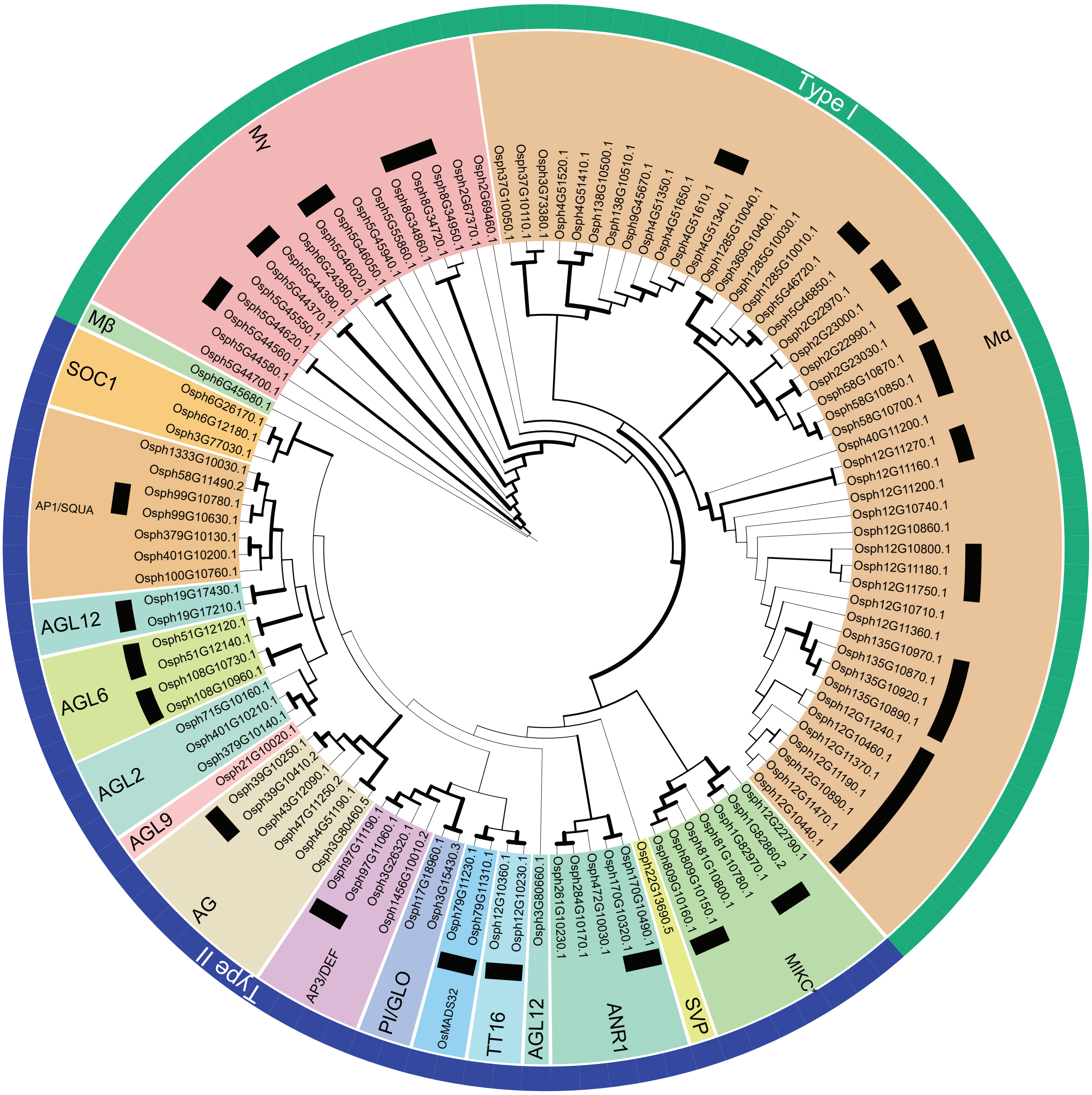

Supplementary Figure 11. Phylogenetic analyses of the MADS-box transcription factor gene family in *Ophrys*

Line thickness indicates bootstrap support. Colours indicate different gene groups, while black bars indicate tandem duplications or duplication within a small chromosome region.

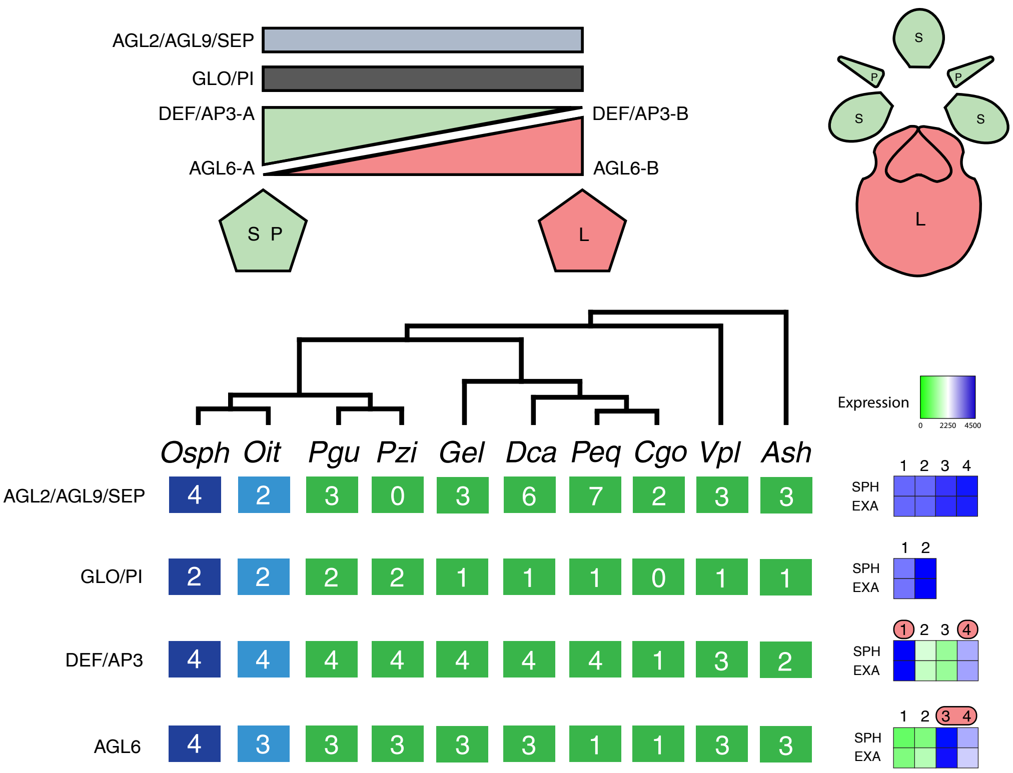

Supplementary Figure 12. MADS-box genes and hypothesised perianth specification in *Ophrys*

According to the perianth code model[66], different protein complexes specify sepal (S) and petal (P) identity (SP complex) or labellum (L) identity (L complex). While *GLO/PI* and *SEP* homologues are expressed throughout the perianth, different *DEF/AP3* and *AGL6* homologues are responsible for S/P and L identity[66]. Gene copy numbers of *SEP*, *PI*, *AP3* and *AGL6* homologues, estimated from orthogroup membership, are indicated for *O. sphegodes* (Osph, dark blue) and other orchid genomes (green; abbreviations as in Supplementary Table 11). Since no genome is available for the most closely related studied species, *Orchis italica* (Oit, light blue), gene copy number here is an estimate from transcriptomic data[67]. The heatmap on the right shows gene expression (TMM, RNA-seq data) in unpollinated mature flower labella for *O. sphegodes* (SPH) and *O. exaltata* (EXA). No gene is significantly differentially expressed at FDR < 0.05. Two *AP3* and two *AGL6* homologues (highlighted) show high or moderately high gene expression in the labellum, consistent with expression patterns in *Orchis italica*.

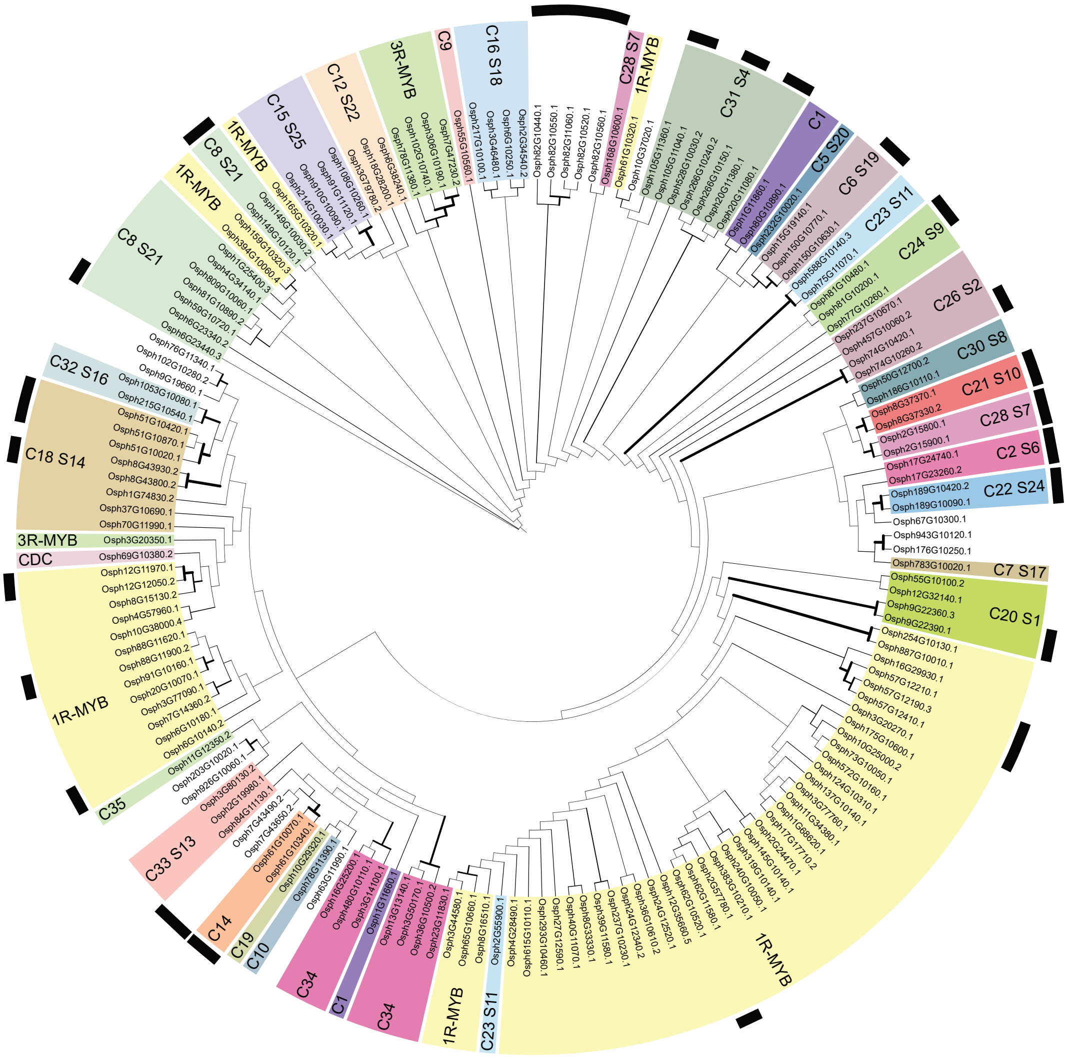

Supplementary Figure 13. Phylogenetic analyses of the MYB transcription factor gene family in *Ophrys*

Line thickness indicates bootstrap support, while black bars indicate tandem duplications or duplication within a small chromosome region. Colours indicate different clades (C; based on conservation of the MYB domain and C-terminal amino acids) and subgroups (S) according to naming for *Arabidopsis thaliana*[68–70]. A total of 89 TFs were identified in the R2R3-MYB (2RMYB) group, which is known to respond to (a)biotic stress and to be involved in many processes including pigmentation, cell shape and development in plants[69,71]. Among the subgroups with function of interest to pollinator attraction in *Ophrys* are C2 S6 involved in anthocyanin biosynthesis[71,72], C28 S7 involved in flavonoid biosynthesis[73], C24 S9 is involved in trichome maturation[68] and C20 S1 is involved in cuticular wax biosynthesis[68].

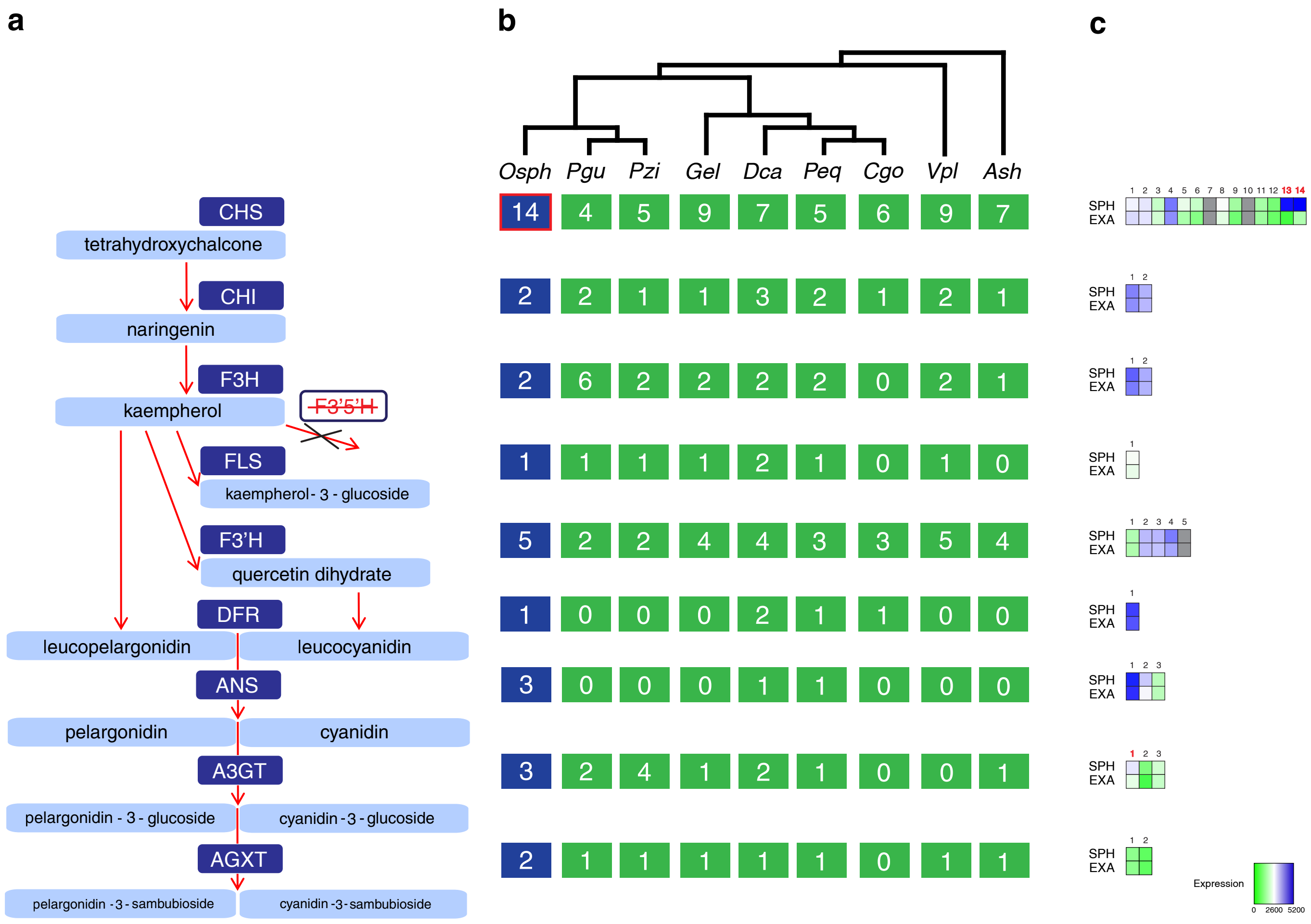

Supplementary Figure 14. Summary of the anthocyanin biosynthetic pathway in *O. sphegodes*

a. Schematic representation of the core pathway showing the biosynthesis of pelargonidins and cyanidins, depicting core proteins in dark blue boxes and metabolites in light blue. In line with a previous study based on transcriptomic data[43], a gene coding for F3'5'H, required for synthesis of delphinidins, does not appear to be present in the genome of *O. sphegodes*. **b.** Gene copy numbers in the *O. sphegodes* (Osph, blue boxes) and other orchid genomes (green boxes), abbreviated by first letter of the genus and the first two letters of the species (as in Supplementary Table 11). Gene copy numbers were estimated by tallying the orchid members of orthogroups containing functionally annotated anthocyanin biosynthetic genes. Gene families expanded in *Ophrys* are shown with a red outline. **c.** Heatmaps of RNA-seq gene expression (TMM, green to blue; grey, not expressed) for *O. sphegodes* gene copies, showing expression for mature flower labella of *O. sphegodes* (SPH) and *O. exaltata* (EXA). Numbers in red indicate significantly differentially expressed genes in a genome-wide analysis (FDR < 0.05).

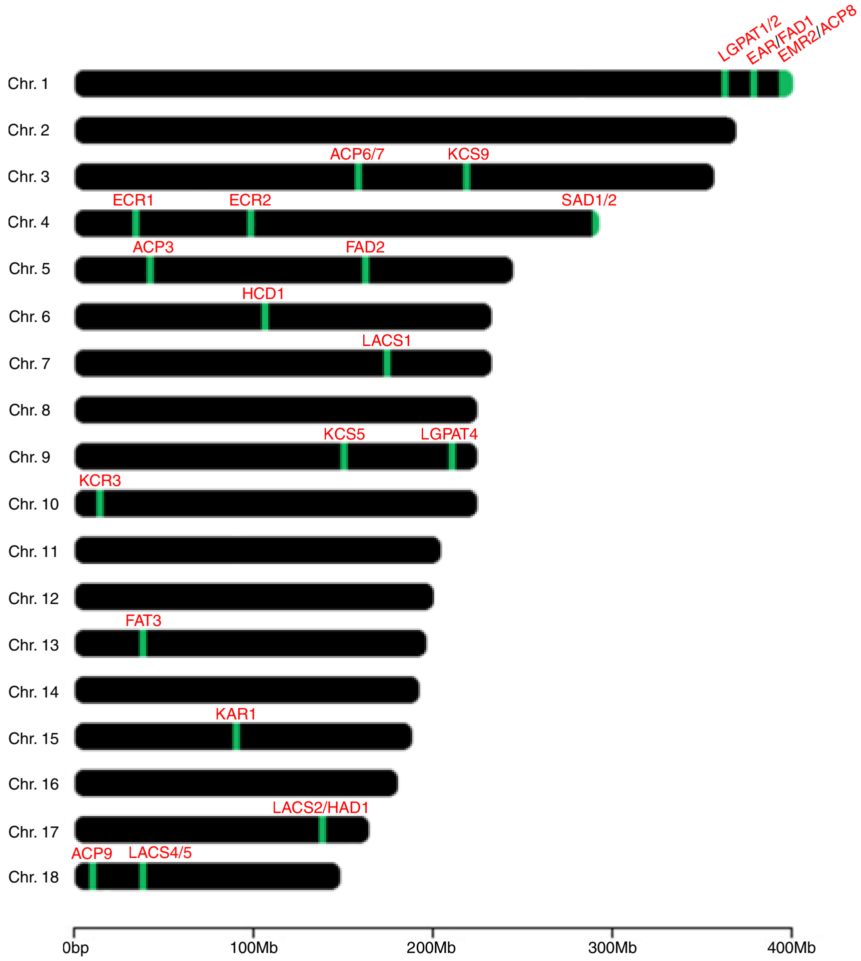

Supplementary Figure 15. Positions of candidate genes

Positions of candidate genes for hydrocarbon biosynthesis and pollinator attraction (Supplementary Tables 7, 8 and Supplementary Data 1) are not clustered in the genome overall but show local gene duplications.

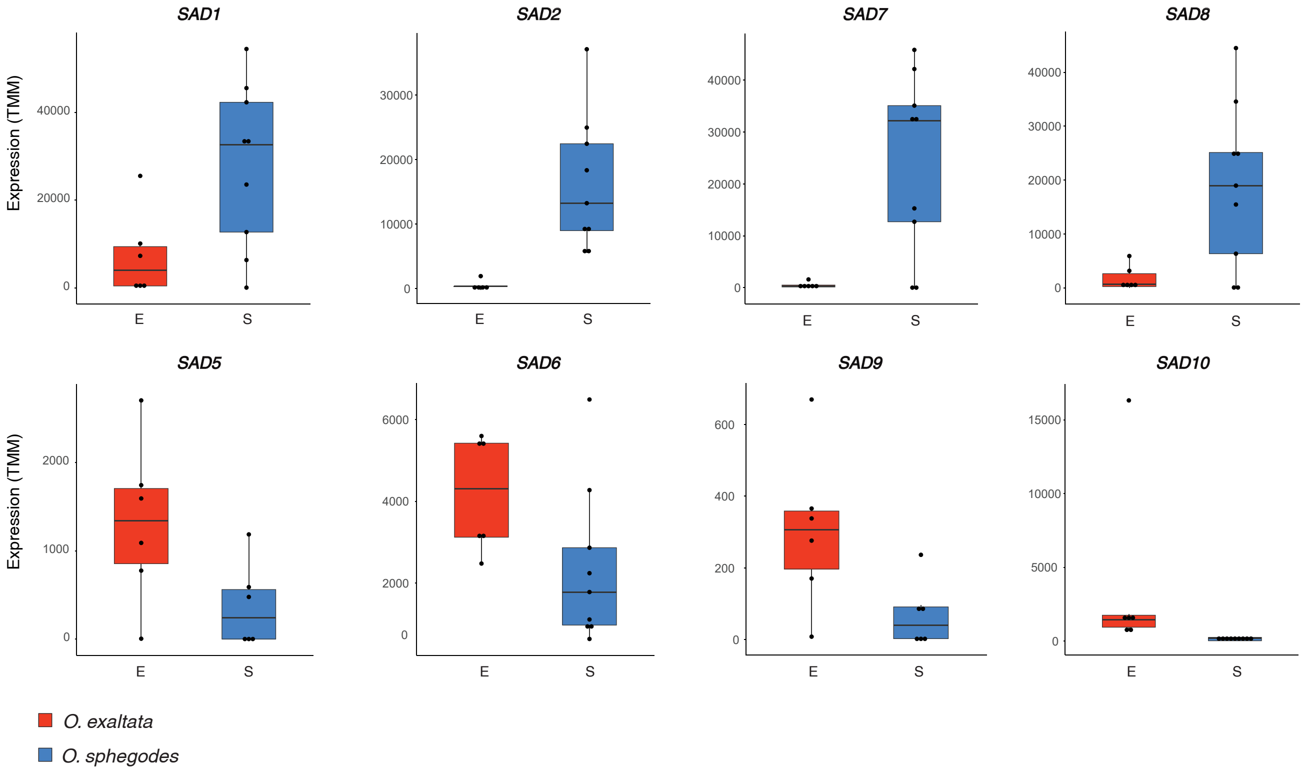

Supplementary Figure 16. Gene expression of pollination-relevant *SAD* genes

Gene expression (TMM) of *SAD2*-type (top row) and *SAD5*-type (bottom row) desaturase transcripts associated with 9- and 12-alkene (*SAD2*) and 7-alkene (*SAD5*) biosynthesis. In line with previous findings[42], *SAD2*-type gene expression is high in *O. sphegodes* (S) and *SAD5*-type gene expression is high in *O. exaltata* (E). Please refer to Supplementary Table 7 for correspondence between the genomic loci and previous *SAD* allele labelling used in ref.[42].

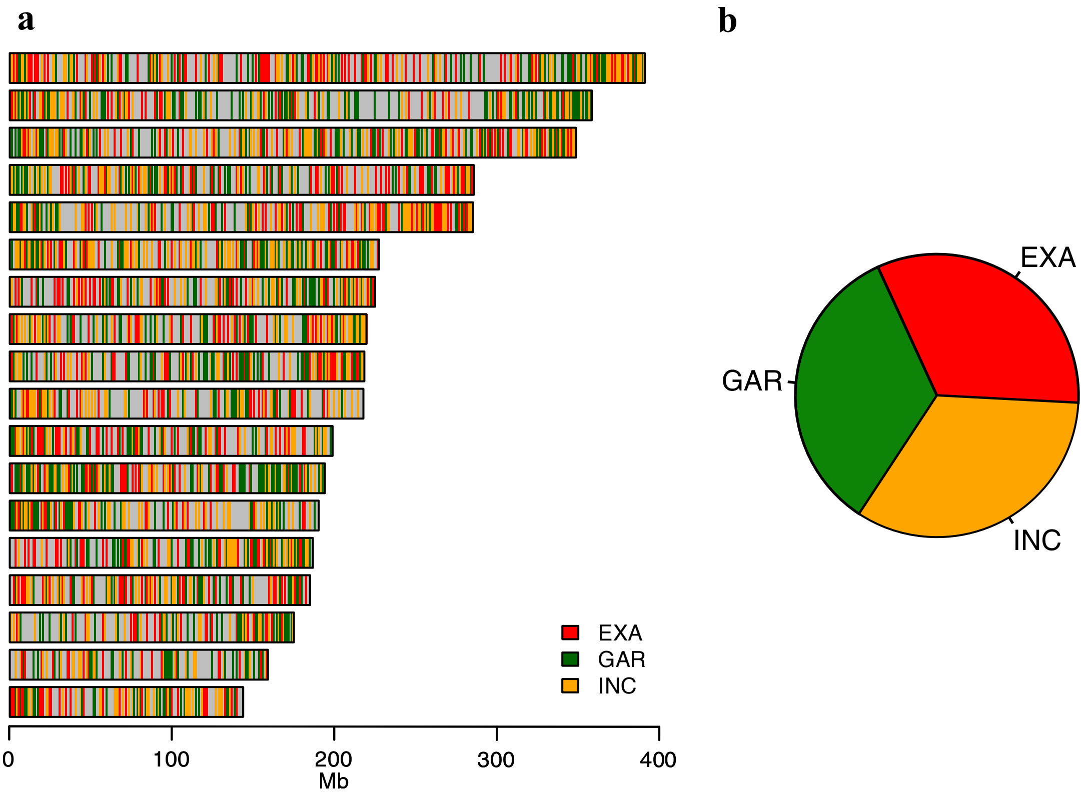

Supplementary Figure 17. Most similar species to *O. sphegodes* per 1 Mb window

a. Most similar species as per chord distance analysis along *O. sphegodes* chromosomes indicated in colour. **b.** Pie chart showing the proportions of genome windows per most similar species. *O. exaltata* (EXA), *O. garganica* (GAR), *O. incubacea* (INC) are shown in red, green, yellow, respectively.

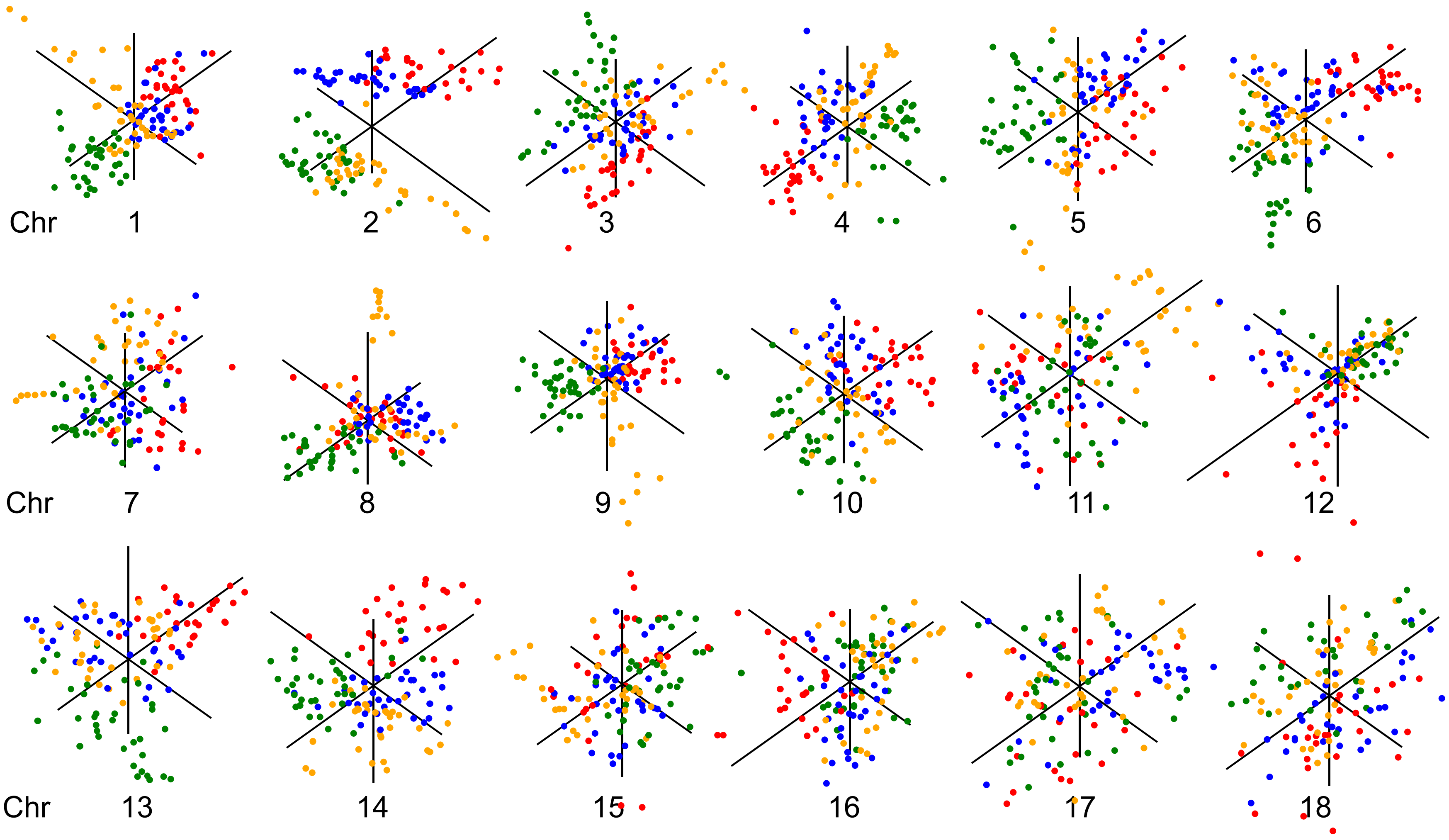

Supplementary Figure 18. GBS PCoA plots for each chromosome

Species are colour coded as in Fig. 5.

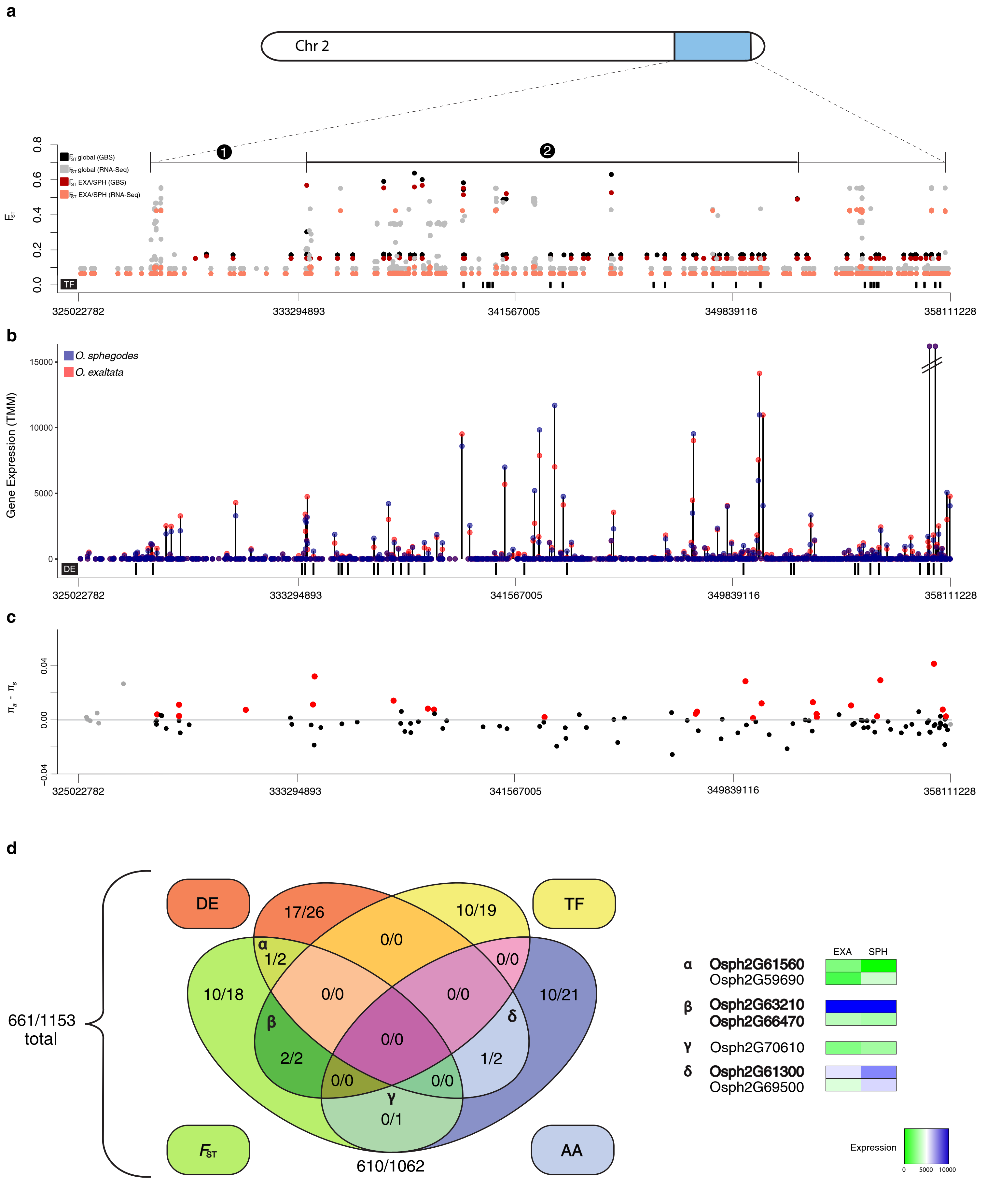

Supplementary Figure 19. ***O. sphegodes*** **chromosome 2 region of differentiation**

a. F_{ST} differentiation along the chromosome, showing 'global' F_{ST} among four species (black, from GBS dataset; grey, from RNA-seq dataset) and pairwise F_{ST} between *O. sphegodes* (SPH) and *O. exaltata* (EXA; dark red, from GBS dataset; light red, from RNA-seq dataset). The lines on top marked 1 and 2 (in black circles) delineate the intervals of high differentiation for the purpose of analysis for RNA-seq and GBS data respectively. Black bars underneath the plot indicate the location of genes annotated as transcription factors (TF). **b**. Gene expression (TMM) for genes in the interval (blue, *O. sphegodes*; red, *O. exaltata*). Black bars underneath the plot indicate genes that are significantly differentially expressed (DE) in a genome-wide analysis (FDR < 0.05). **c**. Differences in non-synonymous (π_a) and synonymous nucleotide diversity (π_s) for genes in the interval (black and red points), excess variation in amino acid (AA) change shown in red (genes with π_a-π_s>0 and greater-than-average π_a). **d**. Venn diagram intersecting sets of genes annotated as TF, significant in DE analysis, with excess AA variation and with elevated F_{ST} (F_{ST} > 0.25). Gene numbers are given for the intervals derived from GBS and RNA-seq data, separated by a forward slash ('/'). Four intersections of set pairs (α, β, γ, δ) contained genes, the identity of which is shown on the right (bold, genes found in the core, GBS-defined, interval). Among these, are an AP2 TF homologue (Osph2G66470, β), a B3-ARF TF (Osph2G63210, β) and a putative ubiquitin conjugating enzyme (Osph2G63100, δ). Annotation information for genes of interest (sets α-δ) is provided in Supplementary Data 7. The total number of genes in the GBS- and RNA-seq-defined intervals are 661 and 1153, respectively. The heatmap on the right shows gene expression (TMM, RNA-seq data) for the genes of interest in unpollinated mature flower labella of *O. sphegodes* (SPH) and *O. exaltata* (EXA).

Supplementary Tables

Supplementary Table 1. Flow cytometry measurements

Flow cytometry data for *O. sphegodes* pollinia ('Sample', haploid) measured on a Cytoflex S machine, Beckman Coulter, using *Solanum lycopersicum* cultivar 'Stupicke polni tyckove rane' as a reference ('Ref') species. Each row represents a different biological *O. sphegodes* replicate. Run1, Run2, Run3 represent three technical replicates. Sample 1C is the average of Run1, Run2, Run3 per biological sample.

Ref 1C	Sample 1C	Interun CV%	Intrarun CV%	Haploid size (Mb)	Run1 1C	Run2 1C	Run3 1C
0.98	4.980	0.82	1.84	4870	5.025	4.970	4.944
0.98	4.914	0.40	1.79	4806	4.933	4.915	4.894
0.98	4.924	0.98	1.88	4816	4.978	4.909	4.885

CV = Coefficient of Variation
1C = DNA amount in picogram of the haploid nucleus

Supplementary Table 2. Statistics of sequencing raw data from Oxford Nanopore (ONT) and Pacific Biosciences (PacBio) platforms

Sequencing platform	Flow cell	Total read number	Total length (Gbp)
**ONT PromethION**	1	5 720 322	90 Gb
**ONT PromethION**	2	1 049 368	24 Gb
**ONT PromethION**	3	4 765 204	88 Gb
**ONT PromethION**	4	9 131 684	126 Gb
**ONT PromethION**	5	1 467 129	23 Gb
**ONT PromethION**	6	3 065 912	53 Gb
**ONT MinION Mk1B**	1	234 692	2.7 Gb
**ONT MinION Mk1B**	2	234 637	1.8 Gb
**PacBio SMRT Cell**	1	266 244	5.9 Gb
**PacBio SMRT Cell**	2	337 031	7.0 Gb
**Total ONT**		25 199 619	409 Gb
**Total PacBio**		603 275	13 Gb

PacBio data were only used for validation, but not for genome assembly.

Supplementary Table 3. Statistics of sequencing raw data from Illumina platforms

Illumina Sequencing Platform	Individual	Library type	Read length (nt)	Files	Total base pairs after trimming
NovaSeq 6000	SPH_8	WGS PE	2 x 250	20190807.B-Alessia_3_R1/R2	328 059 212
NovaSeq 6000	SPH_8	WGS PE	2 x 150	20200128.A-Alessia_3_R1/R2	83 183 820 328
HiSeq 2500	SPH_511B	WGS PE	2 x 125	20170124.A-Sph511B_R1/R2	6 666 301 185
HiSeq 4000	SPH_511B	WGS PE	2 x 150	20170207.A-Sph511B_R1/R2	56 150 212 140
HiSeq 4000	SPH_511B	WGS PE	2 x 150	20170731.B-Sph511B_R1/R2	72 122 655 460
HiSeq 4000	SPH_511B	WGS PE	2 x 150	20171116.A-Sph511B_R1/R2	49 523 800 508
NovaSeq 6000	SPH_2	Hi-C PE	2 x 150	20191216.B-Ophrys_R1/R2	30 885 826
NovaSeq 6000	SPH_2	Hi-C PE	2 x 150	20200520.B-Ophrys_R1/R2	90 929 210
NovaSeq 6000	SPH_2	Hi-C PE	2 x 150	20200527.B-Ophrys_R1/R2	1 021 481 422

WGS, whole genome sequencing; PE, paired-end.

Supplementary Table 4. Summary of the *Ophrys sphegodes* genome assembly

Assembly version	*Osph-v0.7*	*Osph-v1.0*	*Osph-v1.2*	*Osph-v2.3*
**Total contigs**	11 148	8 074	2 511	1 500
**Total length (bp)**	6 429 540 610	5 215 626 102	5 218 768 602	5 219 753 694
**Longest contig (Mb)**	7.3	7.3	51.3	390.7
**N50**	754 kb	908 kb	4.8 Mb	218 Mb
**N75**	402 kb	537 kb	2.6 Mb	159 Mb
**L50**	2 466	1 759	308	10
**L75**	5 385	3 618	672	17

The versions of the *O. sphegodes* genome assembly refer to: v0.7, after polishing but before removal of under-collapsed contigs; v1.0, after removal of under-collapsed heterozygous contigs; v1.2, after Hi-C scaffolding with SALSA; v2.3, final assembly after 3D-DNA and HiC-Hiker step.

Supplementary Table 5. Chromosome lengths in the *Ophrys sphegodes* genome assembly

Chromosome/Contig	Total length (bp)
Chromosome 1	390 740 925
Chromosome 2	358 121 781
Chromosome 3	348 374 665
Chromosome 4	285 498 563
Chromosome 5	237 963 090
Chromosome 6	227 181 086
Chromosome 7	224 791 954
Chromosome 8	219 458 497
Chromosome 9	218 188 670
Chromosome 10	217 594 550
Chromosome 11	198 636 841
Chromosome 12	193 889 286
Chromosome 13	190 071 460
Chromosome 14	186 519 188
Chromosome 15	183 697 217
Chromosome 16	174 855 801
Chromosome 17	158 874 247
Chromosome 18	143 640 109
Scaffold 19	83 227 664
Unanchored contigs (N= 1481)	92 406 681

Supplementary Table 6. Mapping rate of Illumina and PacBio reads against the genome

A paired-end read is considered properly mapped when both mates map to the same region (Properly paired %). For PacBio data, the proportion of primary alignments is shown instead.

Data set	All mapped (%)	Properly paired (%)
20170124.A-Sph511B	98.05	83.13
20170207.A-Sph511B	96.34	82.68
20170731.B-Sph511B	96.63	83.02
20171116.A-Sph511B	96.72	84.90
20190807.B-Alessia_3	99.73	91.87
20200128.A-Alessia_3	99.28	89.65
**Data set**	**Total mapped (%)**	**Primary mapping (%)**
PacBio data (see Supp. Table 2)	98.06	95.07

Supplementary Table 7. *SAD* genes for odour production included in the gene annotation (as described in Supplementary Note 1)

Gene identifier	*Gene/allele name*	Description	Comments
	VLCFA and hydrocarbon biosynthesis		
Osph4G59510.1 / OsphA1798:SAD1-Alt	*SAD1 / SAD1^{Alt}*	(soluble) **s**tearoyl-**A**CP (or acyl-ACP) **d**esaturase	→*SAD1-B*
Osph4G59550.1	*SAD2*		1.14.19.2; 1.14.19.26 / K03921; →*SAD2-A*
Osph5G55220.1 / OsphA241:SAD3	*SAD3$^{\Psi}$ / SAD3*		→*SAD3*
Osph75G10230.1	*SAD4*		1.14.19.2; 1.14.19.26 / K03921; → *SAD4*
Osph250G10230.1	*SAD5$^{\Psi}$*		→*SAD5-A*
Osph210G10040.1 [a]	*SAD6*		→*SAD6-B* [c]
Osph4G59582.1 / OsphA1441:SAD7	*SAD7$^{\Psi}$ / SAD7*		→*SAD1-A*
Osph4G59580.1 / OsphA1441:SAD8P	*SAD8 / SAD8$^{\Psi}$*		→*SAD2-B*
Osph132G10080.1	*SAD9Ψ*		→*SAD5-A*
Osph886G10040.1 [b]	*SAD10*		→*SAD5-B* [c]
Osph1397G10050.1	*SAD11/Ψ*		→*SAD6-B*

[a] gene model for *SAD6*, corresponding to a previously cloned sequence[42], has exons 1 and 2 annotated on the + strand of contig 210 and exon 3 on the - strand; [b] gene model for *SAD10* spans two contigs (contigs 886 and 210), with break in the second intron and with first coding exon on contig 886; [c] These sequences are full-length and without stop codons, but do not appear to be enzymatically active and were hence previously categorised as non-functional[42,74].

Supplementary Table 8. Transcription factors of interest included in the gene annotation (as described in Supplementary Note 2)

Gene identifier	*Gene/allele name*	Description	Comments
Transcription factors of interest			
Osph97G11060.1	*DEF1H1*	**DEF**/AP3 **h**omologue (homologues of *Orchis italica DEF1-DEF4)*[67,75]	MADS→MADS-MIKC
Osph97G11190.1	*DEF1H2*		MADS→MADS-MIKC
Osph1456G10010.1	*DEF2H1*		
Osph1G46670.1	*DEF2H2Ψ*		
Osph3G26320.1	*DEF3H1*		MADS→MADS-MIKC
Osph3G26350.1	*DEF3H2Ψ*		
Osph18G15220.1	*DEF4H1Ψ*		
Osph17G28910.1	*GL3H1Ψ*	*Arabidopsis thaliana* ***GL3*** **h**omologue	bHLH
Osph17G29140.1	*GL3H2Ψ*		
Osph11G31450.1	*LFY1*	*LFY/FLO* [76,77]	
Osph11G31400.1	*LFY2Ψ*		
Osph59G10460.1	*ML1H1*	*Dendrobium crumenatum MYB* ***M****IXTA*-***L****IKE* ***1*** (*MYBML1*) **h**omologue[78]	
Osph59G10560.1	*ML1H2Ψ*		
Osph168G10600.1 / OsphA63:MYB11H1	*MYB11H1*$^{\Psi}$ */ MYB11H1*	*Arabidopsis thaliana* ***MYB11*** **h**omologue	MYB→MYB
Osph2G70710.1	*SPL8H1*	*Oryza sativa* ***SPL8*** **h**omologue	
Osph2G70890.1	*SPL8H2*		
Osph1G68420.1	*SPL9H1*	*Arabidopsis thaliana* ***SPL9*** **h**omologue	
Osph1G68550.1	*SPL9H2*		
Osph6G51030.1	*SPY1*	*Arabidopsis thaliana* ***SPY*** homologue	

Supplementary Table 9. Repetitive content in the *Ophrys sphegodes* genome

Type		Code	Length (bp)	% in the genome [a]
**Class I**				
	LTR/Gypsy	RLG	2 265 079 734	43.39
	LTR/Copia	RLC	1 593 298 782	30.52
	LTR/LTR	RLX	34 618 888	0.66
	LTR/Bel-Pao	RLB	0	0.00
	LTR/Retrovirus	RLR	0	0.00
	LINE/R2	RIR	12 898 808	0.25
	LINE/L1	RIL	17 816 225	0.34
	LINE/RTE	RIT	18 947 522	0.36
**Class II**				
	TIR/CACTA	DTC	28 268 745	0.54
	TIR/Pif-Harbinger	DTH	27 869 444	0.53
	TIR/hAT	DTA	11 632 404	0.22
	TIR/Mutator	DTM	8 833 173	0.17
	TIR/Tc1-Mariner	DTT	105 801	0.00
	Helitron	DHH	20 226 278	0.39
**Simple repeats**			34 196 269	0.66
	**Total**		4 073 792 073	78.06

[a] Total genome length 5 219 753 694 bp

Supplementary Table 10. Repetitive elements in orchids

Type	*Pgu* (%)	*Pzi* (%)	*Ash* (%)	*Dca* (%)	*Peq* (%)	*Gel* (%)
**LTR/Bhikhari**	0.00	0.00	0.00	0.00	0.00	0.00
**LTR/Cassandra**	0.00	0.02	0.00	0.00	0.00	1.49
**LTR/Caulimoviru**	0.02	0.04	0.06	0.04	0.07	0.00
**LTR/Caulimovirus**	0.04	0.07	0.33	0.02	0.07	0.04
**LTR/Copia**	35.75	33.98	4.97	24.70	7.31	6.42
**LTR/Copia(Xen1)**	0.00	0.00	0.00	0.00	0.00	0.00
**LTR/Delta**	0.00	0.00	0.00	0.00	0.00	0.00
**LTR/DIRS**	0.01	0.02	0.00	0.03	0.00	0.01
**LTR/ERV**	0.00	0.00	0.00	0.00	0.00	0.00
**LTR/ERV-Foamy**	0.00	0.00	0.00	0.00	0.00	0.00
**LTR/ERV-Lenti**	0.00	0.00	0.00	0.00	0.00	0.00
**LTR/ERV1**	0.20	0.21	0.10	0.14	0.20	0.02
**LTR/ERV4**	0.00	0.00	0.00	0.00	0.00	0.00
**LTR/ERVK**	0.09	0.08	0.07	0.01	0.03	0.00
**LTR/ERVL**	0.03	0.02	0.00	0.00	0.00	0.00
**LTR/ERVL-MaLR**	0.00	0.00	0.00	0.00	0.00	0.00
**LTR/Foamy**	0.01	0.01	0.00	0.00	0.00	0.00
**LTR/Ginger**	0.00	0.00	0.00	0.00	0.00	0.00
**LTR/Gypsy**	37.48	37.80	11.84	15.18	36.88	48.33
**LTR/Gypsy-Cigr**	0.06	0.04	0.02	0.01	0.02	0.00
**LTR/Gypsy-Troyk**	0.00	0.00	0.00	0.00	0.00	0.00
**LTR/Gypsy-Troyka**	0.00	0.00	0.00	0.00	0.00	0.00
**LTR/Lenti**	0.00	0.00	0.00	0.00	0.00	0.00
**LTR/LTR**	3.49	5.82	5.58	4.17	4.23	4.72
**LTR/Ngaro**	0.00	0.00	0.04	0.00	0.04	0.30
**LTR/Pao**	0.01	0.03	0.03	0.01	0.02	0.02
**LTR/TATE**	0.00	0.00	0.00	0.00	0.00	0.00
**LTR/Viper**	0.00	0.00	0.00	0.00	0.00	0.00
**Total Gypsy/Copia**	73.23	71.78	16.81	39.88	44.19	54.75

Repetitive elements in six orchid species (table adapted from Li, MH. et al. 2022; *Nat. Plants.* 8, 373-388, Supplementary Table 8). Repetitive element percentages for *V. planifolia* are in Hasing, T. et al., 2020; *Nat. Food.* 1, 811-819, Supplementary Table 2. The listed species are: *Pgu* = *Platanthera guangdongensis*; *Pzi* = *Platanthera zijinensis*; *Ash* = *Apostasia shenzenica*; *Dca* = *Dendrobium catenatum*; *Peq* = *Phalaenopsis equestris*; *Gel* = *Gastroda elata*.

Supplementary Table 11. Gene family expansion and contraction across 21 plant species with sequenced genome

Species	Code	Expanded	Contracted
Amborella trichopoda	*Atr*	188	2 744
Ananas comosus	*Aco*	1 136	1 635
Apostasia shenzhenica	*Ash*	672	2 482
Arabidopsis thaliana	*Ath*	2 026	2 029
Asparagus officinalis	*Aof*	1 668	2 335
Brachypodium distachyon	*Bdi*	721	1 025
Cymbidium goeringii	*Cgo*	431	6 228
Dendrobium catenatum	*Dca*	1 817	391
Gastrodia elata	*Gel*	765	2 994
Musa acuminata	*Mac*	4 115	829
Ophrys sphegodes	***Osph***	**3 712**	**756**
Oryza sativa	*Osa*	1 046	504
Phalaenopsis equestris	*Peq*	1 714	529
Phoenix dactylifera	*Pda*	3 653	610
Platanthera guangdongensis	*Pgu*	841	1 969
Platanthera zijinensis	*Pzi*	732	1 368
Populus trichocarpa	*Ptr*	4 640	429
Sorghum bicolor	*Sbi*	945	712
Spirodela polyrhiza	*Spo*	866	3 301
Vanilla planifolia	*Vpl*	3 248	1 568
Vitis vinifera	*Vvi*	907	1 409

Supplementary Table 12. Accession number of orchid genome assemblies used in this study

Species	Assembly type	Assembly size	Annotated genes	Source
Cymbidium goeringii	chromosomes	3.99 Gb	29 556	http://orchidgenome.com/
Dendrobium catenatum	scaffolds	1.11 Gb	29 257	Requested from authors
Dendrobium chrysotoxum	chromosomes	1.37 Gb	30 044	PRJNA664445
Gastrodia elata	scaffolds	1.06 Gb	18 969	PRJCA000931
Gastrodia elata	chromosomes	1.09 Gb	21 115	PRJCA005619
Phalaenopsis equestris	scaffolds	1.13 Gb	29 545	PRJNA262478
Platanthera guangdogensis	chromosomes	4.27 Gb	22 559	Requested from authors
Platanthera zijinensis	chromosomes	4.19 Gb	24 513	Requested from authors
Vanilla planifolia	chromosome-phased	736 Mb [a]	22 026 [a]	PRJNA668740

Source lists NCBI identifiers. [a] haplotype A.

Supplementary Table 13. Statistics of syntenic analysis of *O. sphegodes* (21 462 genes) compared with other orchid genomes

Orchid species 2	*N* genes species 2	*N* collinear gene pairs	*N* collinear blocks	Mean *N* genes per block
Cymbidium goeringii	29 556	1767	153	11.55
Dendrobium chrysotoxum	27 094	4230	208	20.34
Phalaenopsis aphrodite	19 666	3970	191	20.79
Platanthera zijinensis	24 513	5624	148	38.00
Platanthera guangdongensis	22 559	5216	135	38.64
Vanilla planifolia	29 167	3441	337	10.21

Supplementary Table 14. Statistics of sequence variants

Data set [a]	Filtering [b]	Individuals	Genotype calls
WGS	None	8	191 200 928
	Biallelic	8	174 192 143
	biallelic & SNP	8	146 090 706
	biallelic & indel	8	11 010 748
	DP≥10	8	9 567 686
	DP≥10 & biallelic	8	7 961 327
	DP≥20	8	3 485 576
	DP≥20 & biallelic	8	2 874 985
RNA-seq	None	37	13 177 461
	Biallelic [c]	32	12 535 528
	DP≥5 & biallelic [c]	32	439 708
	DP≥10 & biallelic [c]	32	330 449
	DP≥20 & biallelic [c]	32	233 053
GBS [d]	none	126	1 680 249
	biallelic	126	1 621 433
	biallelic & SNP	126	1 356 864
	biallelic & indel	126	120 557
	DP≥5 & biallelic	126	15
	biallelic & (N_A≥6 × 4 pops)	126	18 862
	biallelic & (N_A≥6 × 4 pops) & F_{ST}≥0.001	126	8 541

[a] WGS: whole-genome (re)sequencing data set from this study (2 individuals × 4 species); RNA-seq, raw data from ref.[25]; GBS, raw data from ref.[54].
[b] Filtering applied across all individuals; DP here refers to the VCF SAMPLE/FORMAT DP tag, not the INFO DP tag. N_A refers to the number of allele calls counted from a given population; F_{ST} as calculated by SPA software. '&' denotes a logical AND operator.
[c] applied to data set excluding bud samples pooled from different individuals and samples with unclear population assignment.
[d] using the data set after exclusion of putatively mis-assigned individual 527I.

Supplementary Table 15. Point mutation transition matrices

***RNA-seq data set** (32 × DP≥5); N= 389 561*				
	**A**	**C**	**G**	**T**
**A**	0.0000	0.1112	0.2940	0.1052
**C**	0.1112	0.0000	0.0840	0.2926
**G**	0.2940	0.0840	0.0000	0.1131
**T**	0.1052	0.2926	0.1131	0.0000
***GBS data set**; N= 1 356 793*				
	**A**	**C**	**G**	**T**
**A**	0.0000	0.1271	0.2897	0.0963
**C**	0.1271	0.0000	0.0707	0.2897
**G**	0.2897	0.0707	0.0000	0.1265
**T**	0.0963	0.2897	0.1265	0.0000
***WGS data set** (8 × DP≥10); N= 7 260 426*				
	**A**	**C**	**G**	**T**
**A**	0.0000	0.1314	0.2966	0.0989
**C**	0.1314	0.0000	0.0459	0.2963
**G**	0.2966	0.0459	0.0000	0.1309
**T**	0.0989	0.2963	0.1309	0.0000

Supplementary Table 16. Distance matrix for comparing genotype calls

Pairwise distance matrix for diploid unphased single-locus genotype calls, including partial genotype calls with an allele call missing. Here, `0` and `1` refer to two different alleles (e.g. A and T in a SNP call), whereas `?` denotes a missing allele.

Distance	**0/0**	**0/1**	**1/1**	**0/?**	**1/?**
**0/0**	0				
**0/1**	0.5	0			
**1/1**	1	0.5	0		
**0/?**	0	0.5	1	0	
**1/?**	1	0.5	0	1	0

Supplementary Notes

Supplementary Note 1

Supplementary Table 7 shows a priori candidate genes and pseudogenes identified by homology to *SAD* genes of interest (for a full list of candidates in very long-chain fatty acid or hydrocarbon biosynthesis and selected transcription factors, see Supplementary Data 1). Unique *Ophrys sphegodes* gene identifiers referring to the haploid reference genome are denoted "Osph" followed by chromosome/scaffold number, "G" and gene number (sorted by gene position), "." and a gene/transcript model number. Where a gene was annotated on an alternative haplotig (as identified by Redundans), the identifier refers to "A" and the haplotig number followed by ":" and the gene name. In several cases, two alleles of the same gene were identified. Letters following a gene name in superscript denote allele designation. Genes/alleles ending in Ψ appear pseudogenised (i.e. with at least one stop codon or frame-shift) when aligned to the full-length sequence of a *bona fide* homologue of interest. Where available, the Comments column adds pipeline-based EC and KO annotation information for biosynthetic genes.

Some of the *SAD* loci identified in the *Ophrys* genome correspond to published sequences previously assumed to be alleles (see ref.[42]) rather than paralogues. We here note the correspondence of genomic loci to these previously identified sequences (arrow symbols). The present naming aims to preserve the gene names for functionally characterised *SAD* sequences encoding enzymatically active proteins[41,74].

Supplementary Note 2

Supplementary Table 8 shows a priori candidate genes and pseudogenes identified by homology to selected transcription factors of interest (see also Supplementary Data 1). Unique *Ophrys sphegodes* gene identifiers referring to the haploid reference genome are denoted "Osph" followed by chromosome/scaffold number, "G" and gene number (sorted by gene position), "." and a gene/transcript model number. Where a gene was annotated on an alternative haplotig (as identified by Redundans), the identifier refers to "A" and the haplotig number followed by ":" and the gene name. In some cases, two alleles of the same gene were identified. Letters following a gene name in superscript denote allele designation. Genes/alleles ending in Ψ appear pseudogenised (i.e. with at least one stop codon or frame-shift) when aligned to the full-length sequence of a *bona fide* homologue of interest. Where available, the Comments column adds TF database annotation.

Supplementary References

1. Wick, R. R., et al. Performance of neural network basecalling tools for Oxford Nanopore sequencing. *Genome Biol.* **20**, 129 (2019).

2. De Coster, W., et al. NanoPack: visualizing and processing long-read sequencing data. *Bioinformatics* **34**, 2666–2669 (2018).

3. Li, H. Minimap and miniasm: Fast mapping and de novo assembly for noisy long sequences. *Bioinformatics* **32**, 2103–2110 (2016).

4. Nagarajan, N., et al. Fast and accurate de novo genome assembly from long uncorrected reads. *Genome Res.* **27**, 737–746 (2017).

5. Martin, M. Cutadapt removes adapter sequences from high-throughput sequencing reads. *EMBnet.journal* **17**, 10–12 (2011).

6. Li, H. Aligning sequence reads, clone sequences and assembly contigs with BWA-MEM. *arXiv: Genomics.* doi: 10.6084/M9.FIGSHARE.963153.V1 (2013).

7. Walker, B. J. et al. Pilon: An integrated tool for comprehensive microbial variant detection and genome assembly improvement. *PLoS One* **9**, e112963 (2014).

8. Nagarajan, N. & Pop, M. Sequence assembly demystified. *Nat. Rev. Genet.* **14**, 157–167 (2013).

9. Simpson, J. T. & Pop, M. The theory and practice of genome sequence assembly. *Annu. Rev. Genomics Hum. Genet.* **16**, 153–172 (2015).

10. Li, F. W. & Harkess, A. A guide to sequence your favorite plant genomes. *Appl. in Plant Sci.* **6**, e1030 (2018).

11. Lantz, H. et al. Ten steps to get started in genome assembly and annotation. *F1000Research* **7**, 148 (2018).

12. Roach, M. J., et al. Purge Haplotigs: allelic contig reassignment for third-gen diploid genome assemblies. *BMC Bioinformatics* **19**, 460 (2018).

13. Guan, D. et al. Identifying and removing haplotypic duplication in primary genome assemblies. *Bioinformatics* **36**, 2896–2898 (2020).

14. Ghurye, J., et al. Scaffolding of long read assemblies using long range contact information. *BMC Genomics* **18**, 1–11 (2017).

15. Pryszcz, L. P. & Gabaldón, T. Redundans: An assembly pipeline for highly heterozygous genomes. *Nucleic Acids Res.* **44**, e113 (2016).

16. Grob, S., et al. Hi-C analysis in *Arabidopsis* identifies the *KNOT*, a structure with similarities to the *flamenco* locus of *Drosophila*. *Mol. Cell* **55**, 678–693 (2014).

17. Liu, C. In Situ Hi-C library preparation for plants to study their three-dimensional chromatin interactions on a genome-wide scale. *Methods Mol. Biol.* **1629**, 155–166 (2017).

18. Ghurye, J. et al. Integrating Hi-C links with assembly graphs for chromosome-scale assembly. *PLOS Comput. Biol.* **15**, e1007273 (2019).

19. Durand, N. C. et al. Juicebox provides a visualization system for Hi-C contact maps with unlimited zoom. *Cell Syst.* **3**, 99–101 (2016).

20. Dudchenko, O. et al. De novo assembly of the *Aedes aegypti* genome using Hi-C yields chromosome-length scaffolds. *Science* **356**, 92–95 (2017).

21. Nakabayashi, R. & Morishita, S. HiC-Hiker: A probabilistic model to determine contig orientation in chromosome-length scaffolds with Hi-C. *Bioinformatics* **36**, 3966–3974 (2020).

22. Russo, A. et al. Low-input high-molecular-weight DNA extraction for long-read sequencing from plants of diverse families. *Front. Plant Sci.* **13**, 883897 (2022).

23. Simão, F. A., et al. BUSCO: Assessing genome assembly and annotation completeness with single-copy orthologs. *Bioinformatics* **31**, 3210–3212 (2015).

24. Manni, M., et al. BUSCO update: novel and streamlined workflows along with broader and deeper phylogenetic coverage for scoring of eukaryotic, prokaryotic, and viral genomes. *Mol. Biol. Evol.* **38**, 4647–4654 (2021)

25. Piñeiro Fernández, L. et al. A phylogenomic analysis of the floral transcriptomes of sexually deceptive and rewarding European orchids, *Ophrys* and *Gymnadenia*. *Front. Plant Sci.* **10**, 1553 (2019).

26. Wicker, T. et al. A unified classification system for eukaryotic transposable elements. *Nat. Rev. Genet.* **8**, 973–982 (2007).

27. Smit, A., et al. RepeatMasker Open-4.0. *http://www.repeatmasker.org* (2015).

28. Larkin, M. A. et al. Clustal W and Clustal X version 2.0. *Bioinformatics* **23**, 2947–2948 (2007).

29. Hoff, K. J., et al. Whole-genome annotation with BRAKER. *Methods Mol. Biol.* **1962**, 65–95 (2019).

30. Hoff, K. J. & Stanke, M. Predicting genes in single genomes with AUGUSTUS. *Curr.*

Protoc. Bioinforma. **65**, e57 (2019).

31. Brůna, T., et al. GeneMark-EP+: eukaryotic gene prediction with self-training in the space of genes and proteins. *NAR Genomics Bioinf.* **2**, lqaa026 (2020).

32. Buchfink, B., et al. Fast and sensitive protein alignment using DIAMOND. *Nat. Methods* **12**, 59–60 (2014).

33. Keilwagen, J. et al. Using intron position conservation for homology-based gene prediction. *Nucleic Acids Res.* **44**, e89–e89 (2016).

34. Haas, B. J. et al. Improving the *Arabidopsis* genome annotation using maximal transcript alignment assemblies. *Nucleic Acids Res.* **31**, 5654 (2003).

35. Dobin, A. et al. STAR: ultrafast universal RNA-seq aligner. *Bioinformatics* **29**, 15-21 (2013).

36. Zhang, G. Q. et al. The *Apostasia* genome and the evolution of orchids. *Nature* **549**, 379–383 (2017).

37. Chung, O. et al. A chromosome-scale genome assembly and annotation of the spring orchid (*Cymbidium goeringii*). *Mol. Ecol. Res.* **22**, 1168-1177 (2021).

38. Kawahara, Y. et al. Improvement of the *Oryza sativa* Nipponbare reference genome using next generation sequence and optical map data. *Rice* **6**, 3–10 (2013).

39. Harkess, A. et al. The asparagus genome sheds light on the origin and evolution of a young Y chromosome. *Nat. Commun.* **8**, 1279 (2017).

40. Schlüter, P. M. & Schiestl, F. P. Molecular mechanisms of floral mimicry in orchids.

Trends Plant Sci. **13**, 228–235 (2008).

41. Schlüter, P. M. et al. Stearoyl-acyl carrier protein desaturases are associated with floral isolation in sexually deceptive orchids. *Proc. Natl. Acad. Sci. U. S. A.* **108**, 5696–5701 (2011).

42. Xu, S., et al. The genetic basis of pollinator adaptation in a sexually deceptive orchid. *PLoS Genet.* **8**, e1002889 (2012).

43. Sedeek, K. E. M. et al. Transcriptome and proteome data reveal candidate genes for pollinator attraction in sexually deceptive orchids. *PLoS One* **8**, e64621 (2013).

44. Rice, P., et al. EMBOSS: The European Molecular Biology Open Software Suite. *Trends Genet.* **16**, 276–277 (2000).

45. Dagona, A. G. BioEdit: a user-friendly biological sequence alignment editor and analysis program for Windows 95/98/NT. *Nucleic Acids Symp. Ser.* **41**, 95–98 (1999).

46. Bucchini, F. et al. TRAPID 2.0: a web application for taxonomic and functional analysis of de novo transcriptomes. *Nucleic Acids Res.* **49**, e101–e101 (2021).

47. Van Bel, M. et al. PLAZA 4.0: an integrative resource for functional, evolutionary and comparative plant genomics. *Nucleic Acids Res.* **46**, D1190-D1196 (2018).

48. Zheng, Y. et al. iTAK: A program for genome-wide prediction and classification of plant transcription factors, transcriptional regulators, and protein kinases. *Mol. Plant* **9**, 1667–1670 (2016).

49. Wang, Y. et al. Population genomics and epigenomics provide insights into the evolution of facultative asexuality in plants. *bioRxiv* 2023.07.18.549461 (2023).

50. Chan, P. P., et al. tRNAscan-SE 2.0: improved detection and functional classification of transfer RNA genes. *Nucleic Acids Res.* **49**, 9077–9096 (2021).

51. Nawrocki, E. P. & Eddy, S. R. Infernal 1.1: 100-fold faster RNA homology searches. *Bioinformatics* **29**, 2933–2935 (2013).

52. Simpson, J. T. et al. Detecting DNA cytosine methylation using nanopore sequencing. *Nat. Methods* **14**, 407–410 (2017).

53. Lee, I. et al. Simultaneous profiling of chromatin accessibility and methylation on human cell lines with nanopore sequencing. *Nat. Methods* **17**, 1191–1199 (2020).

54. Sedeek, K. E. M. et al. Genic rather than genome-wide differences between sexually deceptive *Ophrys* orchids with different pollinators. *Mol. Ecol.* **23**, 6192–6205 (2014).

55. Devey, D. S., et al. Friends or relatives? Phylogenetics and species delimitation in the controversial European orchid genus *Ophrys*. *Ann. Bot.* **101**, 385–402 (2008).

56. Paulus, H. & Gack, C. Pollinators as prepollinating isolation factors: evolution and speciation in *Ophrys* (Orchidaceae). *Isr. J. Bot.* **39**, 43–79 (1990).

57. Xu, S. et al. Floral isolation is the main reproductive barrier among closely related sexually deceptive orchids. *Evolution* **65**, 2606–2620 (2011).

58. Kosman, E. & Leonard, K. J. Similarity coefficients for molecular markers in studies of genetic relationships between individuals for haploid, diploid, and polyploid species. *Mol. Ecol.* **14**, 415–424 (2005).

59. Gower, J. C. Some distance properties of latent root and vector methods used in multivariate analysis. *Biometrika* **53**, 325 (1966).

60. Schlüter, P. M. & Harris, S. A. Analysis of multilocus fingerprinting data sets containing missing data. *Mol. Ecol. Notes* **6**, 569–572 (2006).

61. Cavalli-Sforza, L. L. & Edwards, A. W. F. Phylogenetic analysis models and estimation procedures. *Am. J. Hum. Genet.* **19**, 233-257 (1967).

62. Takezaki, N. & Nei, M. Genetic distances and reconstruction of phylogenetic trees from microsatellite DNA. *Genetics* **144**, 389–99 (1996).

63. Bolger, A. M., et al. Trimmomatic: a flexible trimmer for Illumina sequence data. *Bioinformatics* **30**, 2114–2120 (2014).

64. Putri, G. H., et al. Analysing high-throughput sequencing data in Python with HTSeq 2.0. *Bioinformatics* **38**, 2943–2945 (2022).

65. Robinson, M. D., et al. edgeR: a Bioconductor package for differential expression analysis of digital gene expression data. *Bioinformatics* **26**, 139–140 (2010).

66. Hsu, H.-F. et al. Model for perianth formation in orchids. *Nature Plants* **1**, 15046 (2015)

67. Valoroso, M. C., et al. The MADS-box genes expressed in the inflorescence of *Orchis italica* (Orchidaceae). *PLoS One* **14**, e0213185 (2019).

68. He, C. et al. Mining MYB transcription factors from the genomes of orchids (*Phalaenopsis* and *Dendrobium*) and characterization of an orchid R2R3-MYB gene involved in water-soluble polysaccharide biosynthesis. *Sci. Rep.* **9**, 13818 (2019).

69. Fan, H. et al. Genome-wide identification and expression analyses of R2R3-MYB transcription factor genes from two Orchid species. *PeerJ* **8**, e9781 (2020).

70. Dubos, C. et al. MYB transcription factors in *Arabidopsis. Trends Plant Sci.* **15**, 573–581 (2010).

71. Li, B.-J. et al. New insight into the molecular mechanism of colour differentiation among floral segments in orchids. *Commun. Biol.* **3**, 89 (2020)

72. Li, C., et al. Anthocyanin biosynthesis induced by MYB transcription factors in plants. *Int. J. Mol. Sci.* **23**, 11701 (2022).

73. Lau, S. E., et al. dsRNA silencing of an R2R3-MYB transcription factor affects flower cell shape in a *Dendrobium* hybrid. *BMC Plant Biol.* **15**, 1–14 (2015).

74. Sedeek, K. E. M. et al. Amino acid change in an orchid desaturase enables mimicry of the pollinator's sex pheromone. *Curr. Biol.* **26**, 1505–1511 (2016).

75. Aceto, S. et al. The analysis of the inflorescence miRNome of the orchid *Orchis italica* reveals a *DEF*-like MADS-box gene as a new miRNA target. *PLoS One* **9**, e97839 (2014).

76. Montieri, S., et al. Isolation of the *LFY/FLO* homologue in *Orchis italica* and evolutionary analysis in some European orchids. *Gene* **333**, 101–109 (2004).

77. Schlüter, P. M., et al. A screen of low-copy nuclear genes reveals the *LFY* gene as phylogenetically informative in closely related species of orchids (*Ophrys*). *Taxon* **56**, 493–504 (2007).

78. Gilding, E. K. & Marks, M. D. Analysis of purified *glabra3-shapeshifter* trichomes reveals a role for *NOECK* in regulating early trichome morphogenic events. *Plant J.* **64**, 304–317 (2010).
