# Supplementary material for: Genome of the early spider-orchid Ophrys sphegodes provides insights into sexual deception and pollinator adaptation
Source: Nat Commun. 2024 Jul 26;15:6308. doi: 10.1038/s41467-024-50622-4 (PMC11282089; doi:10.1038/s41467-024-50622-4)
Supplement: Supplementary file 3 — Description of Additional Supplementary Files [file 41467_2024_50622_MOESM3_ESM.pdf]

Description of Additional Supplementary Files

File Name: Supplementary Data 1
Description: Candidate genes for odour production included in the gene annotation

File Name: Supplementary Data 2
Description: Additional candidate genes identified from annotation data

File Name: Supplementary Data 3
Description: List of transcription factors identified

File Name: Supplementary Data 4
Description: List of protein kinases identified

File Name: Supplementary Data 5
Description: Summary of genes with elevated F_{ST} on chromosome 2

File Name: Supplementary Data 6
Description: GO enrichment analysis for differentiated chr 2 region

File Name: Supplementary Data 7
Description: Summary of annotation information for genes of interest identified on chromosome 2
