# Supplementary material for: Genome of the early spider-orchid Ophrys sphegodes provides insights into sexual deception and pollinator adaptation
Source: Nat Commun. 2024 Jul 26;15:6308. doi: 10.1038/s41467-024-50622-4 (PMC11282089; doi:10.1038/s41467-024-50622-4)
Supplement: Supplementary file 11 — Reporting Summary [file 41467_2024_50622_MOESM11_ESM.pdf]

nature portfolio

Corresponding author(s): Alessia Russo, Philipp Schlüter

Last updated by author(s): Jul 1, 2024

Reporting Summary

Nature Portfolio wishes to improve the reproducibility of the work that we publish. This form provides structure for consistency and transparency in reporting. For further information on Nature Portfolio policies, see our Editorial Policies and the Editorial Policy Checklist.

Statistics

For all statistical analyses, confirm that the following items are present in the figure legend, table legend, main text, or Methods section.

n/a	Confirmed	
☐	☒	The exact sample size (*n*) for each experimental group/condition, given as a discrete number and unit of measurement
☒	☐	A statement on whether measurements were taken from distinct samples or whether the same sample was measured repeatedly
☐	☒	The statistical test(s) used AND whether they are one- or two-sided<br>*Only common tests should be described solely by name; describe more complex techniques in the Methods section.*
☒	☐	A description of all covariates tested
☒	☐	A description of any assumptions or corrections, such as tests of normality and adjustment for multiple comparisons
☐	☒	A full description of the statistical parameters including central tendency (e.g. means) or other basic estimates (e.g. regression coefficient) AND variation (e.g. standard deviation) or associated estimates of uncertainty (e.g. confidence intervals)
☐	☒	For null hypothesis testing, the test statistic (e.g. *F*, *t*, *r*) with confidence intervals, effect sizes, degrees of freedom and *P* value noted<br>*Give P values as exact values whenever suitable.*
☒	☐	For Bayesian analysis, information on the choice of priors and Markov chain Monte Carlo settings
☒	☐	For hierarchical and complex designs, identification of the appropriate level for tests and full reporting of outcomes
☒	☐	Estimates of effect sizes (e.g. Cohen's *d*, Pearson's *r*), indicating how they were calculated

Our web collection on statistics for biologists contains articles on many of the points above.

Software and code

Policy information about availability of computer code

Data collection	No software was used for data collection
Data analysis	Raw data basecalling: Guppy v3. Genome assembly: Miniasm v0.3 and Minimap2 v2.17. Genome correction: Pilon v1.23 and Racon v1.4.3. Heterozygosity correction: Redundans v0.11. Methylation analysis: Guppy v5.0.11, Nanopolish v0.13.3. HiC scaffold: Salsa v2.3, 3D-DNA pipeline v-180114. Genome annotation: BRAKER2 v2.1.6 (that included AUGUSTUS v3.4.0, ProtHint v2.6.0, GeneMark-EX v4.64), GeMoMa v1.8; PASA v2.5.1, AHRD v3.3.3, TRAPID v2.0, tRNAscan-SE v2.0.9, Barrmap v0.9. Repetitive elements annotation and analysis: RepeatModeler v2.0.1, LTR_harvest v2.0.0, EMBOSS package v6.6.0. Phylogenomic analysis: OrthoFinder v2.5.4, RAxML v8.2.11, TreePL v1.0, BEAST" v2.7.0, CAFE v5.0.0, TranslatorX v1.1 (part of MitoPhAST-master v3.0 package), MUSCLE v5.1.0. Population genetics: BWA-MEM v2.2.1, freebayes v1.0.2, samtools v1.10, SPA v0.1, BayeScan v2.1.

For manuscripts utilizing custom algorithms or software that are central to the research but not yet described in published literature, software must be made available to editors and reviewers. We strongly encourage code deposition in a community repository (e.g. GitHub). See the Nature Portfolio guidelines for submitting code & software for further information.

Data

Policy information about availability of data

All manuscripts must include a data availability statement. This statement should provide the following information, where applicable:

- Accession codes, unique identifiers, or web links for publicly available datasets
- A description of any restrictions on data availability
- For clinical datasets or third party data, please ensure that the statement adheres to our policy

The genome assembly and raw sequencing data generated for this study, including ONT data, PacBio data, Illumina WGS and HiC data, were submitted to NCBI under BioProject number PRJNA994461 [https://www.ncbi.nlm.nih.gov/bioproject/PRJNA994461]. The Whole Genome Shotgun project has been deposited at DDBJ/ENA/GenBank under the accession JBANGT000000000 [https://www.ncbi.nlm.nih.gov/nuccore/JBANGT000000000]. The version described in this paper is version JBANGT010000000 [https://www.ncbi.nlm.nih.gov/nuccore/JBANGT010000000]. The RNA-seq data used for genome annotation and expression analysis can be found on NCBI under accession PRJNA57427920 [https://www.ncbi.nlm.nih.gov/bioproject/?term=PRJNA574279]. The GBS data used for population genetic analysis can be found in the NCBI accession PRJNA25733113 [https://www.ncbi.nlm.nih.gov/bioproject/?term=PRJNA257331]. Annotation data, including protein-coding gene annotation, transposable element database and annotation, non-coding RNA, as well as the alternative haplotig fasta file can be found on figshare under doi: 10.6084/m9.figshare.25398166 [https://doi.org/10.6084/m9.figshare.25398166]. Transposable element sequences were also deposited in the TREP database (https://trep-db.uzh.ch/).

Research involving human participants, their data, or biological material

Policy information about studies with human participants or human data. See also policy information about sex, gender (identity/presentation), and sexual orientation and race, ethnicity and racism.

Reporting on sex and gender	*Use the terms sex (biological attribute) and gender (shaped by social and cultural circumstances) carefully in order to avoid confusing both terms. Indicate if findings apply to only one sex or gender; describe whether sex and gender were considered in study design; whether sex and/or gender was determined based on self-reporting or assigned and methods used. Provide in the source data disaggregated sex and gender data, where this information has been collected, and if consent has been obtained for sharing of individual-level data; provide overall numbers in this Reporting Summary. Please state if this information has not been collected. Report sex- and gender-based analyses where performed, justify reasons for lack of sex- and gender-based analysis.*
Reporting on race, ethnicity, or other socially relevant groupings	*Please specify the socially constructed or socially relevant categorization variable(s) used in your manuscript and explain why they were used. Please note that such variables should not be used as proxies for other socially constructed/relevant variables (for example, race or ethnicity should not be used as a proxy for socioeconomic status). Provide clear definitions of the relevant terms used, how they were provided (by the participants/respondents, the researchers, or third parties), and the method(s) used to classify people into the different categories (e.g. self-report, census or administrative data, social media data, etc.) Please provide details about how you controlled for confounding variables in your analyses.*
Population characteristics	*Describe the covariate-relevant population characteristics of the human research participants (e.g. age, genotypic information, past and current diagnosis and treatment categories). If you filled out the behavioural & social sciences study design questions and have nothing to add here, write "See above."*
Recruitment	*Describe how participants were recruited. Outline any potential self-selection bias or other biases that may be present and how these are likely to impact results.*
Ethics oversight	*Identify the organization(s) that approved the study protocol.*

Note that full information on the approval of the study protocol must also be provided in the manuscript.

Field-specific reporting

Please select the one below that is the best fit for your research. If you are not sure, read the appropriate sections before making your selection.

☐ Life sciences ☐ Behavioural & social sciences ☒ Ecological, evolutionary & environmental sciences

For a reference copy of the document with all sections, see nature.com/documents/nr-reporting-summary-flat.pdf

Ecological, evolutionary & environmental sciences study design

All studies must disclose on these points even when the disclosure is negative.

Study description	The study includes the genome assembly of the orchid Ophrys sphegodes, comparative genomic analyses including published data (21 genomes in total), as well as population genetic studies between O. sphegodes and other three sympatric Ophrys species, O. exaltata, O. garganica and O. incubacea. For the genome assembly, one individual was used. A further individual was used for polishing and for HiC. Raw data for the population genetic study were from a previous publication and re-analysed, and include GBS data from 127 individuals of the aforementioned four Ophrys species.

Research sample	Genome assembly: one individual of Ophrys sphegodes (Orchidaceae) cultivated at the University of Napoli and originally derived from a natural population in South Italy. Leaf tissues were used for DNA extraction.<br>Comparative genomics: our genome assembly + 20 previously published plant genomes.<br>Population genetic study: previously published GBS data from O. sphegodes, O. exaltata, O. garganica, O. incubacea from the same geographic area as the plant used for genome assembly.
Sampling strategy	One Ophrys sphegodes individual was chosen for DNA extraction and sequencing of the reference genome.
Data collection	DNA was extracted from leaves of Ophrys sphegodes, and sequenced on PromethION platform. Fast5 data was basecalled to retrieve the fastq necessary for the assembly. For HiC, chromatin was crosslinked, digested, and religated. Final religated samples were sequenced on Illumina platform.
Timing and spatial scale	Genome assembly: leaf tissue was harvested in January 2018 at the University of Naples, Italy, and flash frozen. DNA extraction and sequencing were performed in Zürich, Switzerland, between September and December 2018. HiC: leaf tissue was crosslinked in January 2018, ground to powder, and stored at -80°C in Naples, Italy. Tissue powder was processed to a final HiC sample in September 2019 in Zürich, Switzerland, a HiC library prepared and sequenced.
Data exclusions	No data were excluded from the overall analysis. The filtering strategy for gene annotation and population genetic analysis are described in detail in SI. For pop gen, one GBS sample out of 127 was excluded from analysis due to unclear species assignment (see SI).
Reproducibility	The genome reference is that of a single, unique individual. There was no disagreement between independent sequence data sets of this individual. For genome size estimation, three independent pollinia from the same reference individual were used.
Randomization	Not relevant to this study, since a single genome reference was produced.
Blinding	This study involves no parts in which observer bias could have influenced the assignment of samples to different groups. Species identity of previously published data sets was known and not part of this study.

Did the study involve field work? ☐ Yes ☒ No

Reporting for specific materials, systems and methods

We require information from authors about some types of materials, experimental systems and methods used in many studies. Here, indicate whether each material, system or method listed is relevant to your study. If you are not sure if a list item applies to your research, read the appropriate section before selecting a response.

Materials & experimental systems

n/a	Involved in the study	
☒	☐	Antibodies
☒	☐	Eukaryotic cell lines
☒	☐	Palaeontology and archaeology
☒	☐	Animals and other organisms
☒	☐	Clinical data
☒	☐	Dual use research of concern
☐	☒	Plants

Methods

n/a	Involved in the study	
☒	☐	ChIP-seq
☐	☒	Flow cytometry
☒	☐	MRI-based neuroimaging

Dual use research of concern

Policy information about dual use research of concern

Hazards

Could the accidental, deliberate or reckless misuse of agents or technologies generated in the work, or the application of information presented in the manuscript, pose a threat to:

No	Yes	
☒	☐	Public health
☒	☐	National security
☒	☐	Crops and/or livestock
☒	☐	Ecosystems
☒	☐	Any other significant area

Experiments of concern

Does the work involve any of these experiments of concern:

No	Yes	
☒	☐	Demonstrate how to render a vaccine ineffective
☒	☐	Confer resistance to therapeutically useful antibiotics or antiviral agents
☒	☐	Enhance the virulence of a pathogen or render a nonpathogen virulent
☒	☐	Increase transmissibility of a pathogen
☒	☐	Alter the host range of a pathogen
☒	☐	Enable evasion of diagnostic/detection modalities
☒	☐	Enable the weaponization of a biological agent or toxin
☒	☐	Any other potentially harmful combination of experiments and agents

Plants

Seed stocks	No seed stock was used, Ophrys sphegodes being a long-lived, herbaceous perennial. The reference individual is part of the living collection of plants at the University of Napoli (S.C.)
Novel plant genotypes	No novel plant genotypes were produced.
Authentication	The reference individual is taxonomically identified as Ophrys sphegodes Mill. and phenotypically consistent with other such plants in Gargano, South Italy.

Flow Cytometry

Plots

Confirm that:

☒ The axis labels state the marker and fluorochrome used (e.g. CD4-FITC).

☒ The axis scales are clearly visible. Include numbers along axes only for bottom left plot of group (a 'group' is an analysis of identical markers).

☒ All plots are contour plots with outliers or pseudocolor plots.

☒ A numerical value for number of cells or percentage (with statistics) is provided.

Methodology

Sample preparation	A pair of pollinia from Ophrys sphegodes was crushed with Otto I buffer, transferred to a Petri dish and co-chopped with 2x2cm tissue from reference leaf Solanum lycopersicum. The suspension was filtered, mixed with Otto II buffer, and stained with propidium iodide in the dark at 4°C for 1 hour.
Instrument	Cytoflex S, Beckman Coulter
Software	CytExpert2.3
Cell population abundance	We used analytical flow cytometry, therefore we do not have information about sorted populations. In our measurement, the reference 2C population abundance was 5.78% and the Ophrys 1C population abundance was 53.78%.
Gating strategy	We used 3 different gating strategies. 1. The nuclei population was gated in SSC-A vs. Fluorophhore intensity area (PerCP-A). 2. The singlets were gated using PerCP area vs. PerCP-Width as events with linear relationship between these parameters. 3. We gated out all the events during first 100 seconds of the run, to allow the sample to stabilize.

☒ Tick this box to confirm that a figure exemplifying the gating strategy is provided in the Supplementary Information.
